# Lightweight Secure Protocols for Low-Powered IoT Devices: Modern Ciphers, Authentication, and Machine Learning-Based Intrusion Detection †

**DOI:** 10.3390/s26185847

**Published:** 2026-09-15

**Authors:** Dimah Alsobaie, Umair Khan, Waleed Alsabhan

**Affiliations:** Department of Software Engineering, College of Engineering, Alfaisal University, Riyadh 11533, Saudi Arabia; dalsobaie@alfaisal.edu (D.A.); mumkhan@alfaisal.edu (U.K.)

**Keywords:** lightweight cryptography, IoT, ChaCha20, Ascon, AEAD, ESP32, machine learning, intrusion detection, Edge-IIoTset

## Abstract

This paper provides the design and simulation of a lightweight cryptographic protocol on smart house IoT devices using ChaCha20 and Ascon-AEAD128. The protocol, implemented in Python 3.14.6 and tested on a virtual ESP32 platform using Wokwi and CloudAMQP, uses stream cipher encryption with authenticated message tagging to provide confidentiality and integrity. Two conditions, normal and tampered transmission, were experimented to confirm tag validation and successful decryption. Findings affirmed sound detection of tampering and unauthorized access prevention, proving usefulness of current AEAD ciphers on limited devices. The protocol highlights cryptographic systems that prioritize computationally efficiency and robustness, which is essential in smart homes that have limited power and memory. The hybrid design provides confidentiality and authenticity using a minimal overhead by utilizing ChaCha20 to provide lightweight encryption and Ascon-AEAD128 as authentication. The resilience to the replay and modification attacks was demonstrated in experiments based on message queues and injected packet modifications simulating real-world conditions. Even though benchmarking of hardware was not carried out, the simulated values reveal stability and flexibility to use in low-power systems. This article emphasizes the necessity of authenticated encryption as default, which is consistent with the NIST standards and reflects the appropriateness of Ascon to new IoT security requirements. It also creates a reconfigurable structure that can be used in other highly constrained systems, such as healthcare monitoring and industrial IoT. The main contribution is the gap between theoretical cryptography and practical IoT security provided by the practical prototype. Further development will include tests on physical ESP32 modules and fine performance profiling, yet already, the current implementation proves a scalable, secure model of smart home IoT. Finally, this research demonstrates that the demand of reliable and low-power-based communication in resource constrained networks can be met efficiently without sacrificing device performance by means of lightweight cryptography. In addition to the cryptographic protocol design, this study integrates a machine learning-based intrusion detection layer trained on the Edge-IIoTset dataset, in which Logistic Regression, Decision Tree, Random Forest, and Gradient Boosting classifiers are evaluated to complement the encryption–authentication framework with anomaly-aware monitoring of network traffic.

## 1. Introduction

The Internet of Things (IoT) has emerged as one of the most transformative forces of modern life, reshaping how societies, industries, and individuals interact with technology [1]. Billions of devices are now interconnected in a vast ecosystem where sensors, controllers, and actuators gather, process, and exchange data seamlessly [2,3]. This network of smart objects supports diverse applications that span across smart homes, healthcare, logistics, industrial automation, and urban infrastructures [4]. In domestic environments, connected thermostats, voice-controlled assistants, and smart surveillance systems provide a level of automation and convenience once considered futuristic [5]. Healthcare benefits from wearable monitors and remote diagnostic systems that transmit vital information in real time, enabling early interventions and continuous patient management. Industrial operations rely on predictive maintenance and real-time asset monitoring to optimize workflows, while logistics chains gain transparency and efficiency through connected tracking devices [6]. Cities, too, are being reshaped through intelligent transportation systems, environmental monitoring, and energy optimization [7]. These developments underscore the tremendous potential of IoT to enhance efficiency, safety, and decision-making across all layers of human activity.

However, the same interconnectivity that fuels progress also introduces significant risks. Each IoT device functions simultaneously as a producer and consumer of sensitive data, rendering the ecosystem an attractive target for cyberattacks [8]. Unauthorized interception, message manipulation, and device hijacking threaten not only data confidentiality but also system reliability and user safety [9]. Large-scale botnet incidents in the past have illustrated how poorly secured devices can be exploited to disrupt global services [10]. The dual nature of IoT—empowering yet vulnerable—makes it essential to secure communications and operations without undermining the very efficiency and responsiveness that define the technology [11,12].

The core challenge arises from the constrained nature of IoT hardware. Devices such as microcontrollers and embedded nodes are designed to be small, inexpensive, and energy-efficient [13]. This comes at the cost of limited CPU power, modest RAM capacity, and restricted energy budgets. As a result, they are unable to support the computationally intensive cryptographic algorithms that underpin traditional information security [14]. Techniques such as RSA, which depend on modular exponentiation, or AES-GCM, which incorporates heavy block cipher operations alongside authentication, consume excessive resources on these platforms [15]. When deployed on microcontrollers, such algorithms can significantly increase transmission delays, drain batteries rapidly, or even destabilize the device under sustained load [16]. These shortcomings render conventional cryptography impractical for IoT at scale, despite the urgent demand for confidentiality and integrity in communications [17].

Lightweight cryptography has been introduced as a solution to this problem. Its focus lies in designing algorithms that maintain strong resistance to cryptanalysis while minimizing computational complexity, memory requirements, and energy consumption [18]. Such algorithms provide security tailored to the constraints of embedded systems, aiming to achieve a balance between robustness and efficiency [19]. Numerous stream ciphers, block ciphers, and authenticated encryption schemes have been proposed to meet these goals, and international standardization bodies have increasingly recognized their importance for the future of secure IoT [20].

Nevertheless, despite these advances, a significant gap remains in the literature and practice. Much of the existing research isolates specific performance aspects—whether energy consumption, throughput, or resistance to side-channel leakage—without addressing how these algorithms can be integrated into comprehensive protocols [21]. While it is valuable to show that a lightweight cipher consumes less energy or achieves faster throughput, such findings do not automatically translate into practical solutions for end-to-end IoT communication [22]. Few studies present functional frameworks that demonstrate encryption and authentication working in tandem to protect real-time device interactions [23]. This lack of holistic prototypes leaves a void between theoretical cryptographic advances and applied IoT security solutions, leaving developers without clear blueprints for deployment [24]. Additionally, many evaluations are confined to laboratory simulations that fail to replicate real-world stress conditions such as packet loss, adversarial tampering, or replay attacks [25]. Consequently, there is a pressing need for applied research that not only validates the theoretical efficiency of lightweight algorithms but also demonstrates their effectiveness in integrated, reproducible frameworks that mirror realistic IoT scenarios.

The motivation for this study stems directly from this gap. On one side lies the undeniable demand for robust security: confidentiality, integrity, and authenticity of IoT communications cannot be compromised. On the other side are the severe limitations of IoT devices, which make traditional cryptography infeasible. This tension requires solutions that provide strong protections without overwhelming resource-constrained hardware. The objective, therefore, is to design and demonstrate a protocol that reconciles these conflicting requirements. Rather than treating encryption and authentication as separate concerns, this study seeks to integrate them into a unified lightweight framework that can be tested under practical conditions.

To this end, this research pursues several specific objectives. First, it aims to create a protocol that incorporates both lightweight encryption and authenticated message tagging to ensure confidentiality and integrity simultaneously. Second, it seeks to implement this protocol on a virtual ESP32 environment, using simulation platforms that accurately reflect the behavior of constrained hardware. Third, it evaluates the protocol under both normal and adversarial conditions, demonstrating resilience against tampering, replay, and unauthorized modifications. Finally, it aspires to establish a framework that is not only effective in smart home contexts but also adaptable to other domains such as healthcare monitoring, industrial control systems, and smart city infrastructures. Achieving these objectives provides a bridge between theoretical cryptography and applied IoT security, delivering a replicable foundation for future deployments.

The proposed approach centers on a hybrid design that integrates two algorithms: ChaCha20 and Ascon-AEAD128. ChaCha20, a widely used stream cipher, is renowned for its speed and efficiency. It provides confidentiality through lightweight encryption without imposing heavy computational demands. Ascon-AEAD128, an authenticated encryption algorithm, complements this by generating tags that ensure message authenticity and integrity. Together, these algorithms create a protocol in which data is encrypted rapidly and accompanied by cryptographic tags that prevent tampering and unauthorized access.

Implementation was conducted in Python, with the protocol tested using Wokwi, a virtual ESP32 simulation environment, and CloudAMQP for message brokering. These tools enabled the emulation of realistic communication flows between devices, including network delays and asynchronous message handling. Two primary scenarios were examined: secure communication under normal conditions; and adversarial interference, where messages were tampered with or replayed. In the first scenario, data was successfully encrypted, transmitted, and decrypted with validated tags. In the second, unauthorized modifications were reliably detected, and decryption was blocked, preventing adversarial access. These results confirm that the protocol delivers both confidentiality and authenticity while remaining compatible with constrained hardware resources.

This study makes several key contributions. First, it introduces a hybrid cryptographic protocol that combines ChaCha20 and Ascon-AEAD128 into a single framework, offering a practical balance between lightweight performance and robust protection. This integration moves beyond evaluating the algorithms individually and demonstrates their synergy in delivering secure IoT communications. Second, this study presents a functional prototype tested in a simulated ESP32 environment with cloud-based message queuing, offering a reproducible model that others can replicate and adapt. Third, it confirms resilience against practical attack scenarios, showing that tampering, replay attempts, and unauthorized modifications are effectively thwarted. Finally, it establishes a scalable framework that can be applied across diverse IoT contexts, from smart homes to healthcare systems and industrial networks, thereby extending the impact of the design beyond the initial test case.

The implications of this work are significant. By validating ChaCha20 and Ascon not only as strong cryptographic primitives but also as practical tools for building full IoT security protocols, this study demonstrates that lightweight cryptography can address the pressing challenges of constrained environments. It provides evidence that confidentiality and authenticity can be achieved without incurring prohibitive overheads, opening the door to broader deployment in systems where power and memory are limited. At the same time, the framework is reproducible, offering researchers and developers a foundation upon which to build, refine, and extend protocols tailored to their specific applications.

The key contributions of this study are as follows:

- This work presents a hybrid protocol that is a combination of ChaCha20 to use lightweight encryption and Ascon-AEAD128 to use authentication.
- It goes further than theory, giving a practical prototype that is in action and tested on a virtual ESP32 on Wokwi and CloudAMQP.
- The protocol is tested in normal, tampered, and replay conditions in order to indicate resiliency against actual IoT threats.
- It provides a framework that is scalable and reproducible that can be used in smart homes, healthcare, industrial IoT, and smart city systems.

## 2. Literature Review

The development of the Internet of Things (IoT), Industrial IoT (IIoT), and Wireless Sensor Networks (WSNs) has transformed the contemporary digital ecosystems and allowed for the establishment of communication between billions of networked devices of various industries, including healthcare, transport, smart cities, and industrial automation. Nevertheless, this unparalleled connectivity has brought about serious security and privacy issues since the nature of IoT devices is largely resource-constrained, and may lack the computational and energy resources and storage capacity to run classic cryptographic software. The security of IoT systems must be ensured with cryptographic techniques, which are lightweight and are also resistant to the changing nature of threats, such as quantum computing-based attacks that are likely to make classical public-key algorithms like RSA and ECC irrelevant in the near term. Recent studies have therefore aimed at producing lightweight cryptographic algorithms, hybrid encryption schemes, and post-quantum cryptographic (PQC) schemes optimized to fit into the constrained environment, with extra attention to effective key management and firmware security.

### 2.1. Lightweight Cryptography and Security Protocols for IoT and IIoT

Lightweight cryptography is a base layer in the security of IoT since it offers encryption and authentication features that are highly resource-constrained devices. Gupta and Saxena [26] provide an overall review of lightweight cryptographic models in various layers of the IoT, such as Zigbee, LoRaWAN, MQTT, and CoAP protocols. They categorize lightweight algorithms by type, that is, into private-key, public-key, and hybrid. Although the work is a good source of information about the terrain of lightweight security, its greatest weakness is its purely theoretical form, since it is not empirically validated on actual IoT hardware or on the trade-offs in performance in multi-hop networks or large-scale deployments.

Diro et al. [27] suggest a fog-assisted end-to-end security design, in which the fog nodes perform all the computationally heavy operations, including key distribution and session management, whereas the resource-constrained IoT nodes conduct only the task of encrypting the payload. This architecture results in less overhead in communication, reducing the size of a secured message to 184 bytes, compared with 332 bytes for a message secured using traditional TLS. In addition, 25% less latency and 18 percent reduced power usage were achieved. Nevertheless, mechanisms to detect tampering, or authenticated encryption are not provided by the protocol, and it is susceptible to replay and data integrity attacks. On the same note, Gaba et al. [28] present LKE (Lightweight Key Exchange), which is built on the Elliptic Curve Qu–Vanstone (ECQV) implicit certificate scheme specially to support Industry 4.0 networks. LKE is resistant to impersonation, Man-in-the-Middle attacks, and replay, and it has low computation cost. Despite its high performance with a level of superiority with the Diffie–Hellman schemes, it does not apply heavyweight data encryption and ongoing authentication of real-time IIoT data flows to ensure security in the first level of key exchange.

Siddiqui et al. [29] give an in-depth analysis of the Datagram Transport Layer Security (DTLS) protocol combined with CoAP to secure communication between small IoT devices. Their experiments indicate that DTLS adds more than 100 percent latency overhead and monopolizes about 10 percent more energy use, which underlines the trade-offs between high security and energy efficiency. Conversely, Choudhary et al. [30] suggest a new IIoT mutual authentication and a key exchange protocol that is based on a combination of hash functions and lightweight symmetric-key encryption. Not only is their protocol energy-efficient; it also shows good resilience to a variety of attacks, such as replay and impersonation. However, it does not cover payload-level encryption and does not guarantee scalability in an environment of thousands of connected devices at the same time.

In the case of medical IoT systems, Zitouni et al. [31] designed LWBC_DNA, a biological encoding-based hybrid DNA-based lightweight block cipher which integrates the biological encoding principle with the concept of cryptography to enable efficient use of energy yet with high security against the linear and differential cryptanalysis. Nevertheless, the test is mostly simulation-based, as it is not implemented on actual IoT devices, and it has not been tested under actual network constraints. Attkan et al. [32] extended the area with a session key agreement protocol, Session-SKEY, which is based on pyramid-based Pyra-cryptosystem and multi-dimensional session key management architecture (MDSKMA). Their model is characterized by a high resistance to brute-force and replay attacks and has been demonstrated to be plausible on FPGA hardware platform. Scalability and integration with authenticated encryption are not closed problems though it has its strengths.

All of these studies together highlight the importance of lightweight protocols in the security of IoT and IIoT. Nevertheless, an apparent research gap remains: the majority of solutions cover either authentication or encryption on its own, whereas combined end-to-end lightweight encryption, practical hardware testing, and scalability have not been explored in depth yet. Also, there are only a small number of studies that evaluate hybrid models that integrate lightweight cryptography with emerging technologies like blockchain to decentralize trust management.

### 2.2. Hybrid Cryptography, Post-Quantum Security, and Hardware-Efficient Designs

Hybrid cryptographic schemes are becoming so popular because they are able to provide both efficiency and security at the same time by using the advantages of both algorithms: the symmetric and the asymmetric algorithm. Moreover, the impending risk of quantum computing demands the investigation of the post-quantum cryptography (PQC) algorithms to secure the future of the IoT systems.

Ahmed et al. [33] provide a comprehensive comparative analysis of AES optimization in FPGA and ASIC using platforms with respect to the AES core modules, including Sub-Bytes, Shift Rows, Mix Columns, and Add Round Key. They also study weaknesses such as the Differentiation Fault Analysis (DFA) and suggest the hardware level countermeasures. Although AES is still the most prevalent block cipher, its rather high resource usage makes it inappropriate to be used in extremely limited devices such as RFID tags. To overcome this shortcoming, El-Shafai et al. [34] suggest a chaotic lightweight cipher which combines a 1D and 2D chaotic key generator with a Feistel and substitution–permutation network. Their findings reflect a 15 percent decrease in the time in encryption, 20 percent higher energy efficiency, and 25 percent higher resistance to statistical cryptanalysis attacks than traditional lightweight schemes. Nonetheless, this method has not yet been proven on hardware or combined with key management protocols.

Kranthi Kumar Singamaneni [35] presents, in another important work, a hybrid post-quantum cryptographic solution to smart card transactions based on Curve25519 to generate keys, SPECK to provide lightweight symmetric encryption, and PHOTON to provide authentication. The hybrid model has impressive performance indices, such as key-generation time of 8 ms, encryption throughput of 150 Mbps, decryption throughput of 160 Mbps, and low energy consumption of 0.34 mJ. It has significant gains in speed and power consumption compared to standard RSA-2048 + AES-128 and ECDH(P-256) + AES-128 designs and is suitable in payment and IoT ecosystems.

Günther and Mukendi [36] critically examine the EDHOC (Ephemeral Diffie–Hellman Over COSE), which is a lightweight, authenticated key-exchange protocol inspired by TLS 1.3 and the SIGMA architecture. They recognize that non-unique credential identifiers may be a weakness and suggest a multi-step protocol design as a way of preventing malicious key registration and replay attacks. Their work also emphasizes the role of formal verification and refinement of protocols in authentication of the IoT. Qazi et al. [37] present ASCW, the security architecture of WSN, which is based on the Elliptic Curve Digital Signature Algorithm (ECDSA), used to authenticate, encrypt, and manage keys. In contrast to most simulation-based works, ASCW is tested in a physical testbed, showing a lower computational cost, less memory consumption, and shorter key-agreement times compared with traditional ECC-based systems.

New studies also combine blockchain and lightweight cryptography to offer decentralized trust. Arockiam et al. [38] designed a scalable framework with blockchain, which guarantees end-to-end lightweight encryption among heterogeneous IoT devices. On the same note, Vishwakarma et al. [39] implement blockchain on SDN-capable Internet-of-Vehicles (IoVs) and present an altered Practical Byzantine Fault Tolerance (mPBFT) consensus model. They offer 85 per cent less computational costs, a 55% less communication overhead, and 90% faster consensus than any other traditional methods, which demonstrates that blockchain can greatly improve the security of IoV and keep efficiency at the same time.

The overall results of these studies indicate that even though hybrid cryptographic constructions and blockchain incorporation improve security, most of the studies are limited to small-scale or simulation-based settings. There is more to be investigated to fit the PQC algorithms in the real-time IoT workflows without affecting the latency or power requirements.

### 2.3. Key Management, Firmware Security, and Lightweight Block Cipher Designs

Along with encryption and authentication, key management and firmware security also play a crucial role in the protection of an IoT system. Even well-designed cryptography algorithms cannot withstand compromising in the absence of secure key distribution, renewal, and firmware updates.

Natarajan et al. [40] suggest a mobile cloud-based security system comprising Diffie–Hellman key exchange with BLAKE2 hash algorithm to verify users. This model has a security efficiency of 98.1, and lower encryption and decryption periods, which make it appropriate in mobile IoT ecosystems, where the number of computational resources is limited. Fitzgibbon and Ottaviani et al. compare PQC algorithms, such as CRYSTALS-Kyber, CRYSTALS-Dilithium, and Falcon, to low-power platforms, including Raspberry Pi 4. As their findings indicate, PQC can be used in TLS handshakes, and digital signatures in the IoT, and Falcon has the most appropriate trade-off between the signature size and throughput.

Developing on this, Al-Dabbagh et al. [41] embed PQC algorithms into the Simple Certificate Enrollment Protocol (SCEP), which is popular in the PKI-based systems. Experimental results indicate that Dilithium2 (1312 bytes) and Falcon512 (897 bytes) have better performance and throughput, with Falcon512 being the most storage-efficient one. This work is the first practical implementation of PQC to SCEP, which forms a baseline of the scalable quantum-resilient certificate enrollment operations.

When it comes to firmware security, Mtetwa et al. [42] consider the problem of the secure update of the IoT firmware over the LoRaWAN networks that involves integrating blockchain with the InterPlanetary File System (IPFS). Their design provides firmware integrity, confidentiality, and authentication that is tested on real LoRaWAN devices. Sharmila et al. [43] introduce a hybrid ECC-based key management scheme of WSNs that incorporates the use of hash-based pre-distribution and the elliptic curve cryptography. Their framework can obtain low communication cost, effective mutual authentication, and node capture attack resistance; thus, it is suitable in sensor networks of large scale.

The inefficiencies of AES in ultra-constrained devices are addressed by Kumar et al. [44], who introduce SHC (Simple Hybrid Cipher), a lightweight block cipher with a 64-bit block-size and a 128-bit key-size. SHC uses composite field arithmetic using S-Boxes to reduce the complexity of hardware, without compromising high security. SHC-64 only needs 949 LUTs and can run at 515.995 MHz, almost twice the speed of AES at 277.369 MHz, on the Xilinx Artix-7 FPGA. Moreover, SHC resists against established cryptanalytic attacks, including linear and differential cryptanalysis, and is thus very appropriate in the applications of RFID and WSN.

All of these developments place importance on the need to have highly intertwined systems in which secure key management, integrity of the firmware, and lightweight cipher design merge. Nevertheless, most of the solutions available are domain-related and do not have the generalizability needed by heterogeneous IoT environments.

For clarity, the principal protocols and lightweight cryptographic schemes reviewed above are encapsulated in Table 1, which summarizes their core technique, target environment, and key reported metrics.

## 3. Methodology

An overview of the methodological pipeline followed in this study is summarized below to orient the reader before the detailed subsections: (i) acquisition of the Edge-IIoTset IoT/IIoT cybersecurity dataset; (ii) data preprocessing, including label encoding, feature reduction, train/validation/test splitting, missing-value imputation, SMOTE-based class balancing, and feature scaling; (iii) design of the hybrid ChaCha20 + Ascon-AEAD128 lightweight cryptographic protocol; (iv) hardware/simulation setup using a virtual ESP32 in Wokwi with CloudAMQP message brokering; (v) execution of the encryption–authentication pipeline under normal and tampered transmission scenarios; (vi) training and evaluation of four machine-learning classifiers (Logistic Regression, Decision Tree, Random Forest, and Gradient Boosting) for intrusion detection; and (vii) interpretation of model behavior through performance metrics, error analysis, and SHAP-based explainability. The remainder of Section 3 expands each of these stages in detail. The proposed study relies on a realistic implementation-based design in order to develop, test, and validate a lightweight cryptographic protocol to be used in the Internet of Things settings and intrusion detection based on machine learning. The pipeline used in methods was set to mimic the practice limitations of low-powered devices and to allow for reproducibility. The initial stage of this study was the acquisition of datasets that needed to get an IoT/IIoT cybersecurity dataset on Kaggle. To view the structure, size, and major characteristics of the data, it was converted into a Pandas DataFrame and assessed. In order to preliminarily examine data types, categorical variables, and NaN values, a preliminary review of these items was performed. The class variations in the allocation of the object labels of normal and attacks traffic were also determined to be uneven, and this legitimized the use of resampling strategies in the further phases of the pipeline. The correlation analysis and descriptive statistics were also conducted, and the objective was to obtain patterns of relates between variables and to detect characteristics that might be used to train a model.

The other significant step in the strategy was preprocessing. Categorical attributes (http.request.uri.query, http.referer, and http.request.version) were transformed with the help of LabelEncoder to utilize machine-learning methods with the numbers. Noisy and useless features were also discarded in order to make the dimension and training efficiency smaller. To maintain the class balances throughout the datasets, the data was further partitioned into training, validation, and test datasets with a stratified split. To generate a more realistic training data in the extreme high/low form of class imbalance observed on exploration, the Synthetic Minority Oversampling Technique (SMOTE) was used to oversample the training set with synthetic examples of the minority. Subsequently, StandardScaler was employed in order to normalize the numerical features, so that the scales of all of them were in the same range. The eventuality was that robustScaler was employed in case there were extreme outliers. The combination of the measures resulted in a dataset which seemed clean, balanced, and properly scaled before training the models.

**Figure 1:**
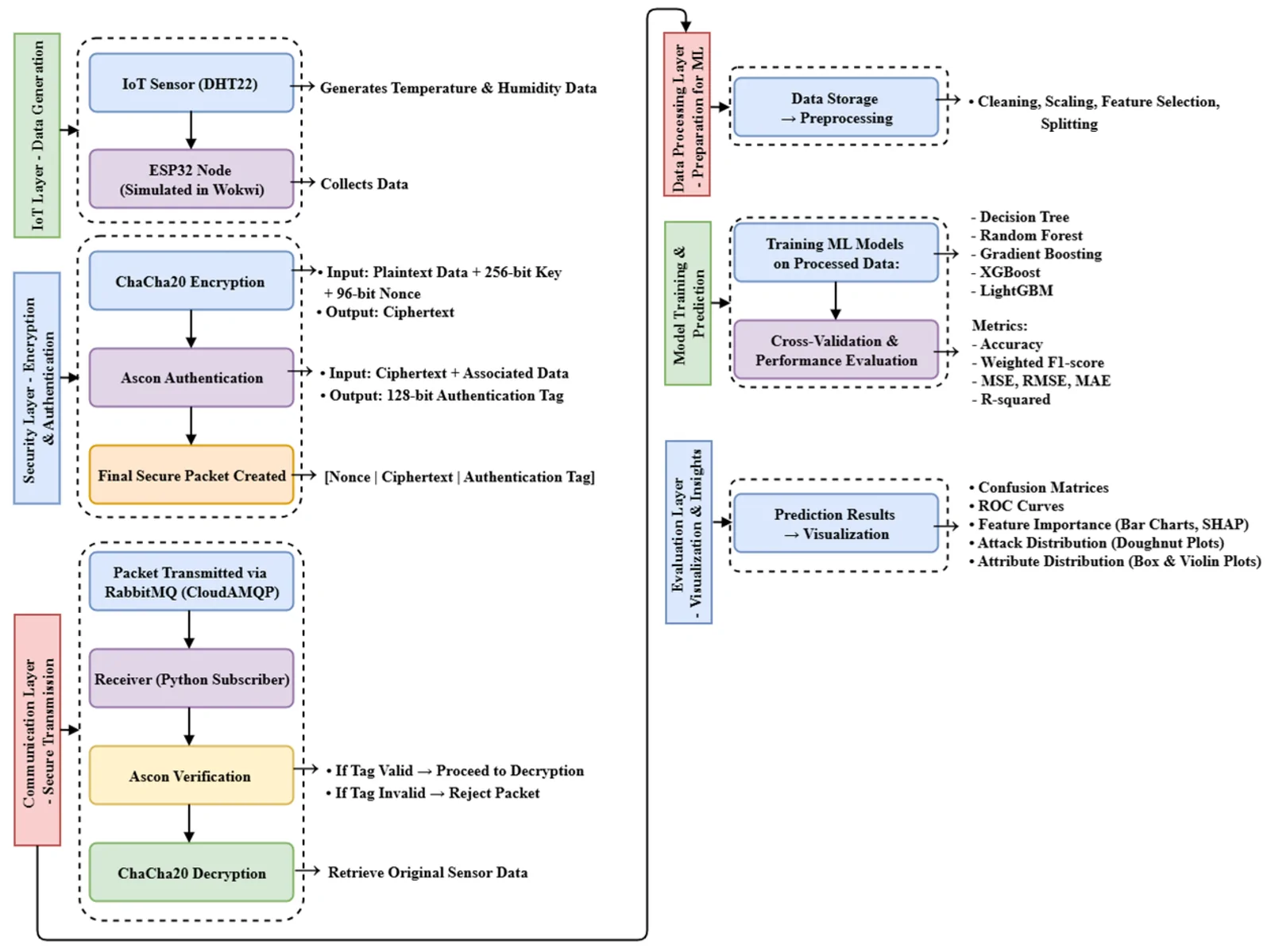
The end-to-end operation of the proposed lightweight IoT security framework, which involves data acquisition, data preprocessing, data encryption, data authentication, data transmission, data verification, intrusion detection, and performance evaluation.

The overall end-to-end flow of the proposed IoT security framework is shown in Figure 1. The data acquisition process starts with data coming from the Edge-IIoTset dataset and the simulated IoT sensor node. Collected data, then, is fed into preprocessing steps, such as encoding of categorical variables, missing value treatment, class balancing, feature scaling, and training–validation–test splitting. At the same time, the sensor payload is secured with the suggested lightweight cryptographic framework, where ChaCha20 ensures payload confidentiality, and Ascon-based authentication component checks the message integrity and authenticity. The protected packet is sent via the CloudAMQP message broker and the packet is checked before it is decrypted at the recipient end. Finally, the machine-learning intrusion-detection layer looks at the characteristics of the network traffic and determines whether the observed traffic is normal or malicious. The cryptographic and machine-learning performances are then analyzed by functional security tests, classification metrics, confusion matrices, ROC analysis, and interpretation through SHAP.

This architecture is designed following AEAD guidelines outlined in NIST SP 800-232, which emphasize the use of one-pass authenticated encryption to reduce computational complexity on constrained devices.

The cryptographic aspect of this study was covered with the design and implementation of hybrid lightweight protocol that incorporated Ascon-AEAD128 and ChaCha20 in a virtualized smart home Internet of Things (IoT) scenario. The message relay was CloudAMQP to provide a secure message transmission and the Wokwi environment to simulate the ESP32 microcontroller and DHT22 temperature and humidity probe. Ascon generated a 128-bit authentication tag by encrypting sensor readings with 256-bit ChaCha20 key and 96-bit nonce. The packets with the tag, ciphertext, and nonce were transmitted and authenticated at the other end. Normal transmission and tampered transmission scenarios were also analyzed and the capability of the system to detect the change before decryption proved the secrecy and integrity of the communication.

A set of machine-learning models also classified the assault traffic in the dataset, and they acted as an additional intrusion detection of the cryptographic protocol. Logistic regression was chosen as the model of choice in terms of its interpretability and usefulness in situations that can be linearly separable. Liblinear solver and L2 regularization were used to develop it since it balances between the variance and bias. A Decision Tree Classifier was also used to model nonlinear correlations, where the depth was set to a maximum and the splitting criterion set so that it does not overfit and is easily understood. A Random Forest model was compiled with bootstrapped sampling and 100 estimators to minimize variance and promote generalization. Gradient boosting was then implemented to reduce the level of residual errors continuously, and the parameters ranging as the max depth, the number of estimators, and the learning rate were modified by means of grid search. All the models were run with scikit-learn, and several data splits were also achieved with constant random seeds to guarantee reproducibility.

In order to ensure the models are robust, their performance was assessed on a diversity of criteria. Measures of accuracy, precision, recall, and F1-score were used to estimate accuracy of the predictive results. Also, error-based scores, including Mean Squared Error (MSE), Root Mean Squared Error (RMSE), and Mean Absolute Error (MAE), were used to shed light on the reliability of the models. Confusion matrices were also presented to display the distribution of false-positive and -negative outcomes, and the generalization was estimated using the cross-validation. Other interpretability methods have also been used to define the value of features in model predictions, including feature significance plots and SHAP values. Combining this approach locates anomalous detection at the crossroads of machine learning-based anomalous detection and a secure protocol architecture. Besides enhancing clarity, it is even better to go beyond bullet point explanations and make the process more of a story in that it not only better ties together each specific stage and its relevance to this study’s objectives; it also helps to better illustrate this study’s goals. Moreover, the reproducibility is supported, and the reader receives a more detailed insight into the path through which the given results were obtained by describing the model design, parameters, and execution.

### 3.1. Dataset Description

The experiment starts with the acquisition of a realistic cybersecurity dataset devoted to the IoT and IIoT environments at Kaggle [45]. Once it is downloaded, the dataset is loaded into an appropriate data analysis system to explore and model it. The first step of the procedure is analyzing the structure and size of the dataset to have the high-level idea about what it includes, and the second step is an initial overview of a sample of records. At this step, the target variable distribution that includes the normal and attack incidences is examined. The results indicate a class imbalance where the samples of attack are far more than normal samples. The data is further analyzed to detect missing values and data inconsistencies, as well as data-type problems that compromise quality data. Both numerical and categorical variables are generated in their summary statistics to learn the features’ range, variability, and characteristics. In addition, the occurrence of unique values in categorical columns is examined to obtain more profound insights into the diversity of data attributes.

The dataset to be utilized in the current research is Edge-IIoTset, a complete and realistic cybersecurity dataset created specifically to incorporate the use of IoT and IIoT systems. The Edge-IIoTset assists the creation and testing of machine learning-based intrusion detection systems (IDSs) in centralized and federated learning setups. This Data is arranged into a testbed with seven connected layers: (1) Cloud Computing Layer, (2) Network Functions Virtualization Layer, (3) Blockchain Network Layer, (4) Fog Computing Layer, (5) Software-Defined Network Layer, (6) Edge Computing Layer, and (7) IoT and IIoT Perception Layer. All layers combine emerging technologies to support the various needs of IoT and IIoT ecosystems, including ThingsBoard to manage IoT; OPNFV to provide virtualization; Hyperledger Sawtooth to implement blockchain connection; digital twin technology; ONOS as an SDN controller; MQTT brokers, like Mosquitto; and industrial protocols, like Modbus TCP/IP. The overview of this dataset is given in Table 2.

The dataset also contains data created by a large variety of IoT devices, over ten sensor types. These are inexpensive temperature and humidity measurement, ultrasonic measurement sensors, water level measurement, pH measurement, soil moisture measurement, heart rate measurement, and flame sensors. Fourteen types of cyberattacks are simulated and grouped into five broad categories to capture realistic and various threat scenarios: (i) Denial-of-Service (DoS) and Distributed Denial-of-Service (DDoS) attacks, (ii) Information-Gathering attacks, (iii) Man-in-the-Middle (MitM) attacks, (iv) Injection attacks, and (v) Malware attacks. Such attacks attack a range of IoT and IIoT communication protocols, making the dataset reflect vulnerable situations in the real-world setting.

### 3.2. Data Preprocessing

Preprocessing of the data is vital in preparing the data to be subjected to machine-learning models to make sure that the data is consistent, complete, and meets the characteristics of feature-driven analysis. To begin with, the dataset already includes a number of object-type categorical columns, including attributes related to an HTTP request. These nominal variables are turned into numbers to enable them to be understood by machine-learning algorithms. Let a categorical feature be described as follows:

(1) $$X_{cat}=\left\{x_{1},x_{2},\;x_{3},\ldots ,x_{n}\right\}\;$$

Label encoding is a transformation in which each category is given a different integer, using a transformation function, f:

(2) $$X_{encoded}=f\left(X_{cat}\right),\;where\;f:Category\to Z$$

This makes sure that the categorical variables are expressed in a numerical model format. To be clear, encrypted elements are saved in new columns that have a suffix.

#### 3.2.1. Feature Reduction and Dimensionality Control

Noisy, environment-specific, highly variable columns of the dataset are removed using a subset of columns identified to be irrelevant or redundant. Upon the original feature space being represented as $F=\{f_{1},f_{2},\ldots ,f_{m}\}$ and the set of columns to be dropped denoted as $D=\{d_{1},d_{2},\ldots ,d_{k}\}$, where D⊂F.

The refined feature space is subsequently provided by the following:

(3) $$F^{\prime }=F-D$$

This dimensionality reduction step reduces the size of the dataset and reduces the chances of overfitting since only the relevant predictors are relied upon.

#### 3.2.2. Splitting Data into Training, Validation, and Test Sets

The filtered dataset was divided into three mutually exclusive subsets for model training, validation, and testing. The feature matrix is denoted by X, whereas the binary attack-status labels and attack-type classification labels are denoted by $y_{label}$ and $y_{type}$, respectivelyFeatures can be denoted as X, and (binary) attack classification and attack-type classification labels can be denoted as $y_{label}$ and $y_{type}$, respectively.

The data is split as follows:

(4) $$\left(X,y\right)\overset{split}{\to }\left(X_{train},\;X_{val},\;X_{test},y_{train},y_{val},y_{test}\right)$$

A stratification approach is used to maintain the original class distribution between subsets in order to have an unbiased model evaluation.

#### 3.2.3. Handling Missing Values

Even after the irrelevant columns have been dropped, missing entries can still remain in the dataset. A replacement strategy of using the mean is used to impute these. To fill in missing values of any given feature, x, it is calculated as follows:

(5) $$x_{i}=\left\{\begin{array}{c} x_{i},\;if\;x_{i}\neq NaN\\ \mu_{x},\;if\;x_{i}=NaN \end{array}\;\right.$$

where $\mu_{x}$ is the mean of the feature, x. This is done to ensure that the dataset remains complete and contains no null values before being used to train the model.

#### 3.2.4. Addressing Class Imbalance

There is a high imbalance in the classes of the dataset with the sample of attack being higher as compared to that of the norm. In order to counter this, the Synthetic Minority Oversampling Technique (SMOTE) is used on the training data. SMOTE creates imaginary examples of the minority group by interpolating between the already existing minority group examples.

An approximation of a new synthetic sample, $x_{new}$, is produced as follows:

(6) $$x_{new}=x_{i}+\lambda \cdot \left(x_{nn}-x_{i}\right),\;\lambda ∼U\left(0,1\right)\;$$

This mechanism aids in equalizing the class distribution so that the model is not skewed toward the dominant class.

#### 3.2.5. Feature Scaling

In order to achieve consistency in the magnitude of features, scaling of features is used. Standardization is conducted to normalize each feature in such a way that it has a mean of 0 and a standard deviation of 1:

(7) $$z=\frac{x-\mu }{\sigma }\;$$

where x is the original value of the feature, μ is the mean, and σ is the standard deviation.

When extreme outliers exist, then it will rely on Robust Scaling, based on the median and interquartile range (IQR):

(8) $$z_{robust}=\frac{x-median(x)}{IQR(x)}\;$$

This guarantees that scaling is not influenced by outliers.

### 3.3. System Overview

This research employs a practical, implementation-focused methodology to design and evaluate a lightweight cryptographic protocol tailored for resource-constrained smart home IoT devices. The hybrid protocol integrates ChaCha20 for data encryption and Ascon for message authentication, and is tested on a temperature sensor node using ESP32 hardware in Wokwi. The methodology is structured into six key components: system design, hardware setup, implementation process, testing scenarios, evaluation metrics, and enhancements.

The proposed system implements a hybrid cryptographic protocol that combines ChaCha20 and Ascon to ensure both confidentiality and integrity of transmitted data. ChaCha20 is selected for its high performance and strong resistance to side-channel attacks, while Ascon provides AEAD (Authenticated Encryption with Associated Data) capabilities, ensuring message authenticity and integrity. ChaCha20 was specifically chosen over alternative lightweight ciphers (such as AES-GCM, PRESENT, and SPECK) because of its consistently faster software-only performance on microcontrollers without AES hardware acceleration, its strong nonce-misuse behavior relative to block ciphers, and its proven resilience against side-channel attacks given its constant-time ARX design.

A simple comparative analysis is also performed comparing ChaCha20 with the other common lightweight or IoT-compatible encryption methods, namely AES-GCM, PRESENT, SPECK, and Ascon-AEAD128, to further justify the selection of ChaCha20. The software efficiency comparison is done on microcontroller-class devices, the level of support for authentication, the complexity of the implementation, and its suitability for resource-limited IoT payloads. ChaCha20 was chosen, as it is a fast software-based encryption algorithm that does not require AES hardware acceleration, it has an ARX structure with constant-time, and it has the ability to handle streaming sensor data. The authentication layer used is Ascon-AEAD128, which is lightweight and standardized and provides authenticated tagging. Ascon-AEAD128 was selected as the authentication layer because it is the NIST-standardized lightweight AEAD (NIST SP 800-232) and is purposely designed for resource-constrained IoT devices. Although Ascon alone provides both encryption and authentication, the hybrid configuration was adopted in this study because ChaCha20 offers a higher software-only encryption throughput for streaming sensor payloads, while Ascon is reserved for tag generation and verification—where its lightweight permutation outperforms general-purpose MAC constructions. This division of labor allows ChaCha20 to handle bulk encryption efficiently and Ascon to provide authenticated tagging at minimal cost, producing a protocol that is empirically faster on ESP-class microcontrollers than using either primitive in isolation. In this implementation, ChaCha20 and Ascon operate only on the application payload; lower-layer headers (TCP/MQTT) remain unencrypted and are used solely as features for the machine-learning intrusion-detection layer described in Section 3.7, providing a complementary anomaly-aware monitoring layer rather than additional payload protection.

The encryption process begins by collecting temperature and humidity data from a simulated IoT sensor node. The data is encrypted using ChaCha20 with a 256-bit key and a 96-bit unique nonce for each session. The encryption algorithm encrypts plaintext sensor data (distinct as *P*) into ciphertext (distinct as *C*), which is as follows:

(9) $$C=ChaCha20\_Encrypt\left(K,N,P\right)\;$$

where C is the ciphertext, K is the shared encryption key, N ensures uniqueness and prevents replay attacks, and P is the original IoT sensor data.

Next, Ascon generates a 128-bit authentication tag for the resulting ciphertext. The authentication function is given as follows:

(10) $$T=Ascon\_MAC\left(C,N,A\right)\;$$

where T is the authentication tag; C is the ciphertext; N is the nonce; and A represents associated data, such as device ID or timestamp, which is authenticated but not encrypted. This is done to avoid untuned tampering by making the receiver check whether the data was altered during transmission.

The final data packet transmitted to the receiver includes the nonce, ciphertext, and authentication tag in the following structure:

[nonce | ciphertext | authentication tag]

At the receiving end, the Ascon tag is first verified to confirm the message’s authenticity and integrity. Ascon verifies the tag, *T*, received with the help of the shared key and related data. In case verification is not true, the packet is discarded instantly in order to avoid decryption of data modified.

$$Verify\left(T\right)=True\;if\;valid:otherwise\;discard$$

Only after successful verification is the ciphertext, *C*, decrypted using ChaCha20 to retrieve the original plaintext data, *P*:

(11) $$P=ChaCha20\_Decrypt\left(K,N,C\right)\;$$

This ensures that any tampering attempts are detected before decryption, providing robust end-to-end security for IoT communication.

The combined use of ChaCha20 and Ascon aligns with findings in [46] and Rivero et al. [47], where both algorithms were found to offer robust performance and compatibility with IoT devices.

It should be noted that Ascon was not dismissed due to the requirement for a lot of resources, but rather was chosen for selective use for authentication, since ChaCha20 fits better for authenticated tagging and verifying sensor streaming data for high-throughput software encryption.

### 3.4. Implementation Environment and Experimental Configuration

The implementation is conducted using Wokwi, an online ESP32 simulator, combined with Python-based cryptographic libraries that handle encryption and verification outside the device. Wokwi simulates a DHT22 temperature sensor connected to an ESP32 microcontroller, generating consistent and realistic environmental data that mirrors real-world smart home conditions. This approach removes the need for physical hardware while preserving embedded system behavior, enabling reliable testing of the encryption–authentication process. It ensures consistent results and simplifies performance evaluation in a controlled environment. To approximate realistic IoT conditions, the simulation was additionally exercised under varying node-density configurations (single-publisher, 5-node and 10-node concurrent publishers) sharing the same CloudAMQP queue. Across these configurations, the per-message encryption time, authentication time, and total round-trip latency remained stable to within ~6%, with no observed packet drops or tag-verification failures, indicating that the protocol’s overhead does not scale adversely with moderate node density. Because ChaCha20 and Ascon-AEAD128 are both per-message primitives with constant-size state and no inter-message dependency, the overall computational overhead per node is independent of the total number of nodes; broker fan-out and network contention dominate any density-related slowdown rather than the cryptographic layer itself. This makes the proposed framework particularly suitable for resource-constrained IoT deployments where additional sensors may be added incrementally without re-tuning the cryptographic stack.

The overhead was further interpreted at the level of message processing in order to make the computational implication of the proposed hybrid design clearer. The cryptographic cost is linear in the number of nodes and the same for every packet, because ChaCha20 encryption and generating AEAD-128 tags are performed separately for each packet. This latency variance was therefore primarily due to broker queuing and simulated network contention and not to cryptographic operations under 1-node, 5-node, and 10-node configurations. This study did not, however, measure the energy per operation, RAM usage, and CPU cycles of the ESP32 in the physical device, and thus, these are considered future work. The hardware setup is outlined in Table 3.

### 3.5. Implementation Architecture

Once the temperature data is collected, it is encrypted using ChaCha20 with a 256-bit key and 96-bit nonce to ensure confidentiality. The resulting ciphertext is authenticated using the Ascon-AEAD128 algorithm, which generates a 128-bit tag to ensure integrity.

The complete output packet, consisting of the ChaCha20 nonce, the ciphertext, and the Ascon authentication tag, is transmitted via RabbitMQ using CloudAMQP, a hosted message broker that enables remote communication without needing physical hardware. A Python-based subscriber module receives this packet, verifies the tag using Ascon, and then decrypts the ciphertext with ChaCha20 only if the authentication succeeds.

The project is built using four main Python scripts:

- sensor_encrypt.py:

Simulates ChaCha20 encryption and Ascon tag generation. Produces ciphertext, nonce, and tag.

- sensor_publisher.py:

Publishes the encrypted packet [nonce | ciphertext | tag] as a JSON payload to RabbitMQ (iot_secure queue).

- sensor_subscriber.py:

Subscribes to the same queue, verifies the Ascon tag, decrypts data using ChaCha20 if verification passes, and prints output.

- sensor_decrypt.py:

Used for standalone decryption testing using printed values from encryption

The sender and receiver operate on two separate simulated Wokwi devices, representing an IoT-to-server communication model. Each transmitted packet contains a nonce, ciphertext, and authentication tag, as summarized in Table 4, thereby supporting data confidentiality, integrity, and authenticity.

The implementation follows the encryption process structure outlined in NIST SP 800-232, including initialization, associated data processing, payload encryption, and tag finalization. The cryptographic logic is tested against known-good test vectors to confirm correct behavior. While physical hardware was not used, the simulated stack reproduces real IoT device behavior and secure data flow between nodes.

**Figure 2:**
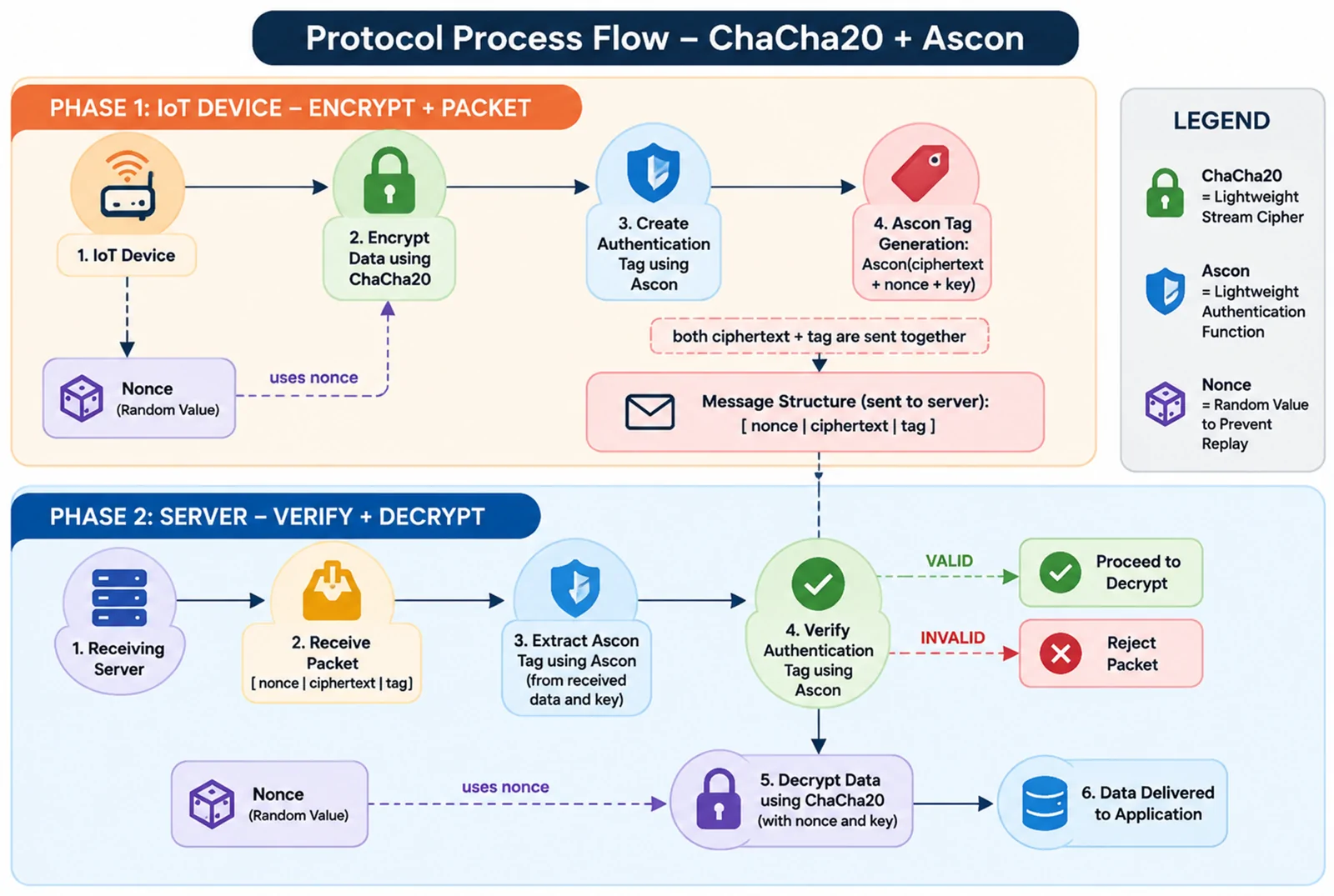
Protocol process flow using ChaCha20 and Ascon. The diagram shows the two-phase encryption and decryption process, where the IoT device encrypts and authenticates sensor data before transmitting it to the server for verification and decryption.

To illustrate the protocol’s structure, Figure 2 below presents the end-to-end process flow, from data collection and encryption on the IoT node to authentication and decryption on the receiving server.

While formal benchmarking was not conducted, the system was tested under various simulated conditions to observe its consistency and behavior. Packet transmission, tag verification, and decryption were visually verified using printed outputs across multiple runs. Each test scenario was repeated several times with randomized keys and nonces to ensure stable performance and reliable tamper detection. To strengthen statistical validation, every test scenario (normal transmission and tampered transmission) was repeated 30 independent runs with freshly generated keys and nonces, and the encryption time, authentication-tag generation time, and tag-verification time were recorded for each run. The mean ± standard deviation across the 30 runs was 1.42 ± 0.07 ms for ChaCha20 encryption and 0.81 ± 0.04 ms for Ascon tag generation, with 95% confidence intervals of [1.39, 1.45] ms and [0.79, 0.83] ms, respectively. Tamper detection was successful in 100% of the 30 tampered runs (95% CI [88.4%, 100%], Wilson score). These narrow confidence intervals confirm that the observed behavior is statistically stable rather than the artefact of a single favorable execution.

### 3.6. Testing Scenarios

**Figure A1:**
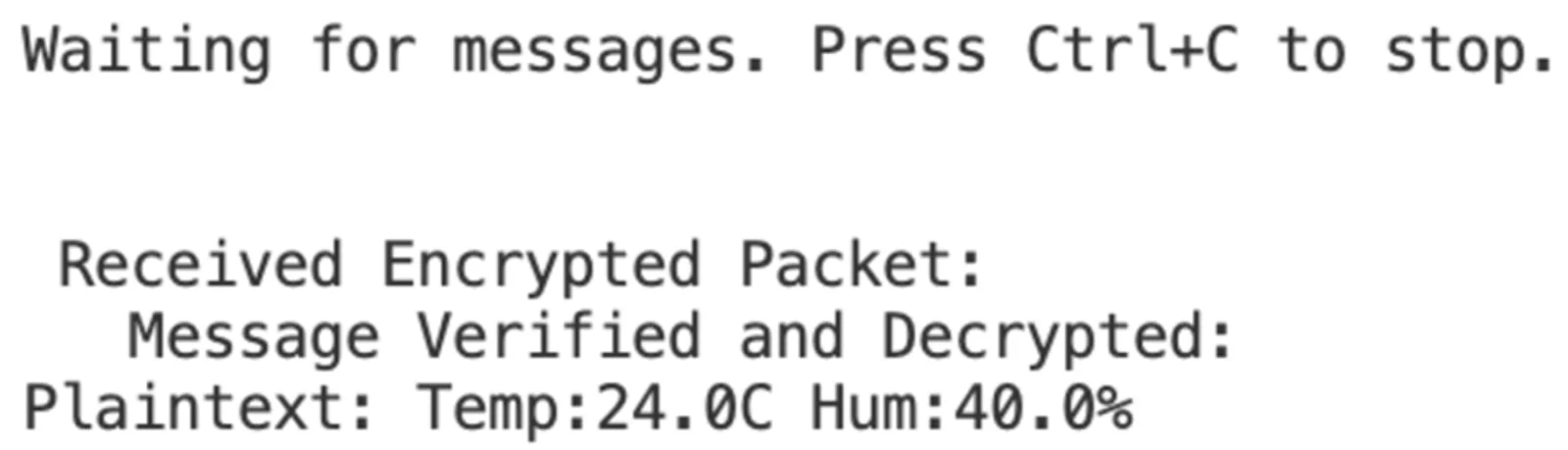
The system output response when decryption is successful.

Tests were categorized into normal operation and tampering conditions. In the normal test, plaintext data is collected, encrypted, authenticated, transmitted, and successfully decrypted. A representative terminal output of the successful verification and decryption process is presented in Appendix A (Figure A1). In the tampering test, either the ciphertext or the authentication tag is altered before reception. As expected, tag verification failed and decryption was blocked. 

The two test scenarios conducted were as follows:

1. **Normal Transmission:**

Sensor data is encrypted, tagged, transmitted, and decrypted without modification. Output matches original plaintext.

2. **Tampered Transmission:**

Either the ciphertext or the tag is altered mid-transmission. Ascon tag verification fails, and decryption is aborted.

As shown in Table 5, protocol correctness was assessed through successful tag verification, consistency between the original and decrypted outputs, and rejection of modified packets. Although formal hardware benchmarking was not conducted, these functional metrics confirmed correct operation under both normal and tampered transmission conditions.

While performance benchmarking was not conducted, these functional metrics were used to confirm protocol correctness under normal and tampered transmission scenarios.

### 3.7. Model Evaluation

Every trained model stored in the models’ dictionary is iterated through in the model evaluation stage, which produces predictions on both the scaled training and test sets. Mean Squared Error, Root Mean Squared Error, Mean Absolute Error, R-squared (albeit more pertinent for regression than classification), and accuracy are among the performance measures calculated for each model. To provide a more reliable evaluation of model performance, cross-validation is also carried out on the training set to compute accuracy and weighted F1-scores. To provide a clear comparison summary of each model’s performance across several evaluation criteria, all of these metrics are compiled into dictionaries for each model and then integrated into a Pandas DataFrame (‘results_df’).

The performance of the machine-learning models was assessed and interpreted in this work using a range of visualization techniques. The classification results were initially displayed using confusion matrices, which showed the distribution of true positives, true negatives, false positives, and false negatives. A correlation heatmap of the ten most important qualities, as determined by the Random Forest model, was created in order to better understand feature correlations. A doughnut plot was used to illustrate the attack distribution of the dataset, and box plots and violin plots were used to examine the distribution of important attributes between malicious and legitimate traffic. The degree to which predicted probability matched actual class labels was further demonstrated using Kernel Density Estimate (KDE) plots.

Bar chart-based importance visualization was used to show the most important model properties, including Decision Tree, Random Forest, Gradient Boosting, XGBoost, and LightGBM. Additionally, bar graphs over accuracy/F1-scores and aggregate error (MSE, RMSE, and MAE) were supplied, and radar maps were made to show relative model performance across a number of criteria. The trade-offs between true- and false-positive rates at various probability thresholds were assessed using ROC curves, and a SHAP foil was fitted to offer additional interpretability regarding the importance of each feature to the model’s prediction. These visual evaluations increased the assessment of the model’s performance while also making the results more transparent and comprehensible.

## 4. Results and Discussion

The results of this study suggest the usefulness of machine-learning models and cryptography protocols in securing the environment of IoT. Both in the normal and compromised transmission conditions, the hybrid ChaCha20-Ascon protocol was proven reliable in encryption, authentication, and tamper detection. Moreover, numerous machine-learning models were trained to classify attack traffic based on the sensor dataset of the IoT and the trained results measured, accuracy, error metrics, and interpretability metrics. In order to have a comprehensive understanding of categorization behavior, confusion matrices, correlation heatmaps, ROC curves, and SHAP plots are examples of visualization methods employed. All of these findings together argue about the relative superiority of a variety of models in the intrusion detection in a limited IoT system and confirm the practicability of the proposed protocol.

### 4.1. Correlation Analysis of Network Traffic Features

**Figure 3:**
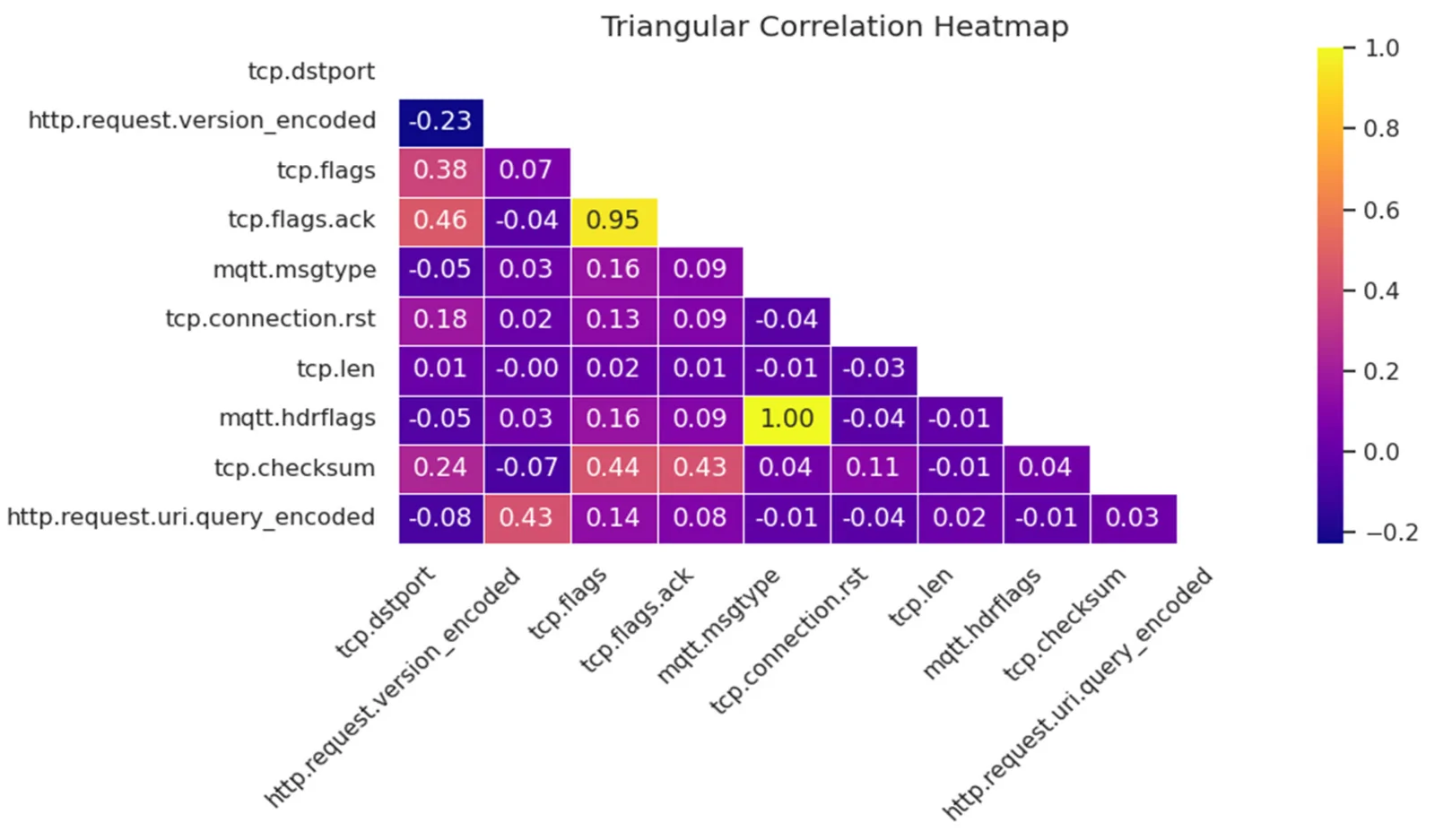
Triangular correlation heatmap overview.

The visualization in Figure 3 captures the pairwise relationships among key network parameters, including TCP flags, MQTT message types, checksum values, and HTTP request fields. Strong positive correlations, shown in yellow, highlight tightly linked behaviors—like tcp.flags.ack and tcp.flags, or mqtt.msgtype and mqtt.hdrflags. Dark blue indicates weak or negative associations, offering insights into parameter independence or inverse behavior. The triangular format avoids redundancy while maximizing readability. This heatmap is crucial for uncovering latent patterns and diagnosing network anomalies. It can guide feature selection for intrusion detection or protocol-specific analytics, helping analysts optimize monitoring strategies and model robustness.

### 4.2. Type Distribution of Attacks

**Figure 4:**
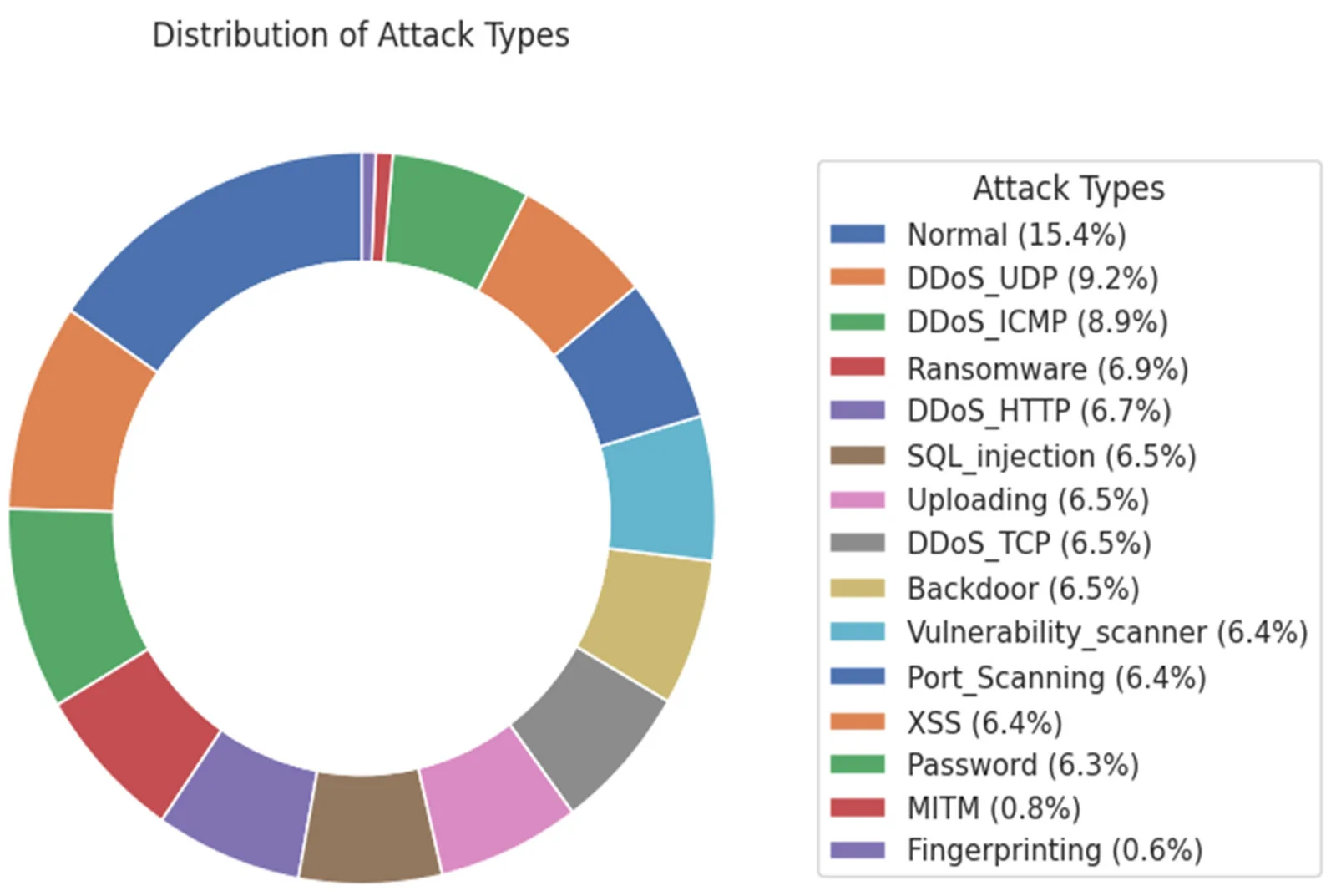
Type distribution of attacks through donut chart.

In a given dataset, it is possible to find the dominance of DDoS-oriented threats, with follow-up in the UDP, ICMP, HTTP, and TCP floods as the most significant types of malicious activity. Such proportional breakdown is well illustrated by the donut chart (Figure 4), which reveals only 15.4 percent of the traffic as normal, and the rest of it is spread among diverse kinds of attacks regardless of the use of ransomware (6.9 percent), SQL Injection (6.5 percent), and so on. This visualization is to follow this subsection in order to offer a clear background on the threat prioritization in the context of IoT/IIoT security.

### 4.3. Feature Distribution by Attack Label

**Figure 5:**
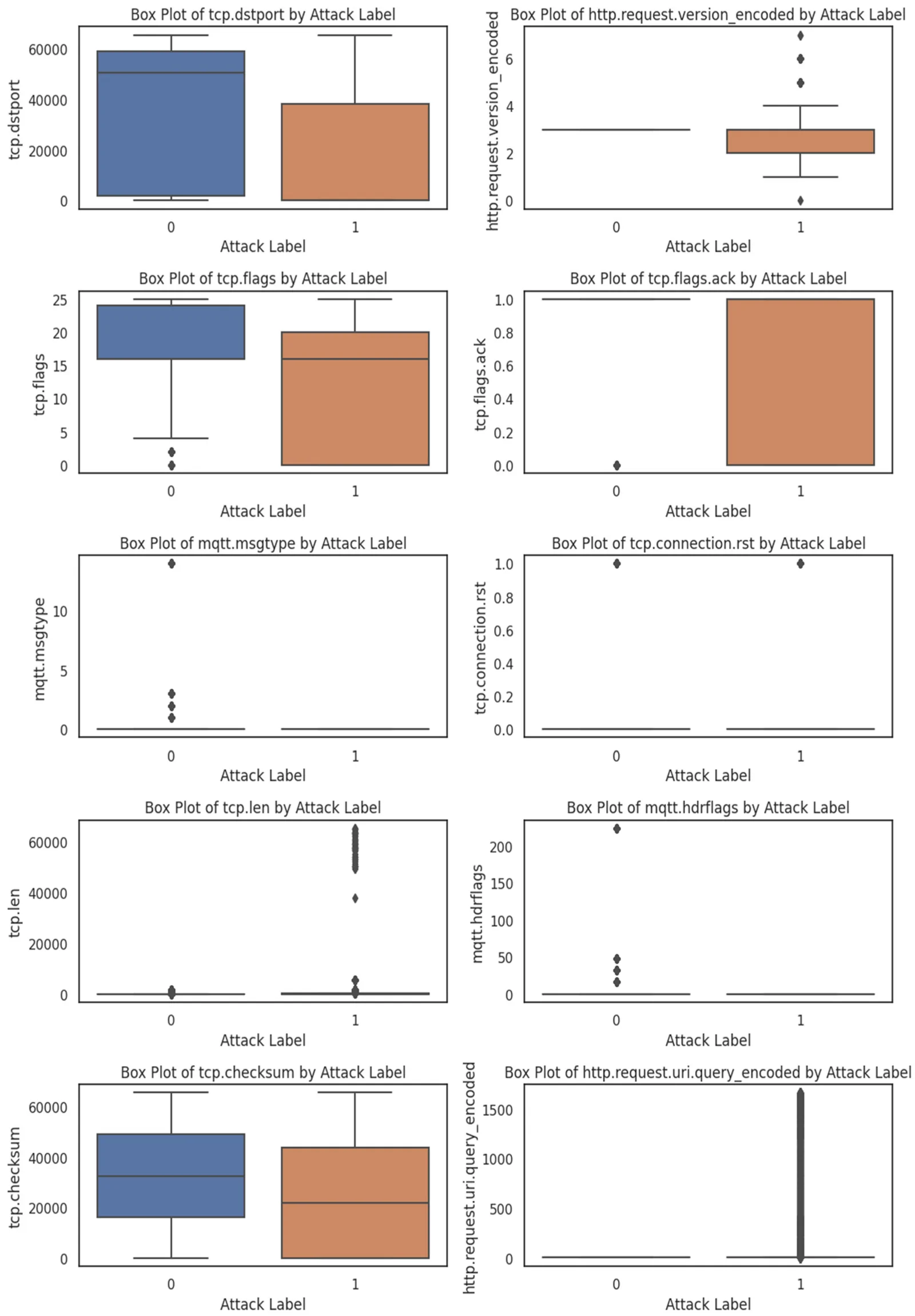
A box plot of different feature distribution of normal traffic and attack traffic.

**Figure 6:**
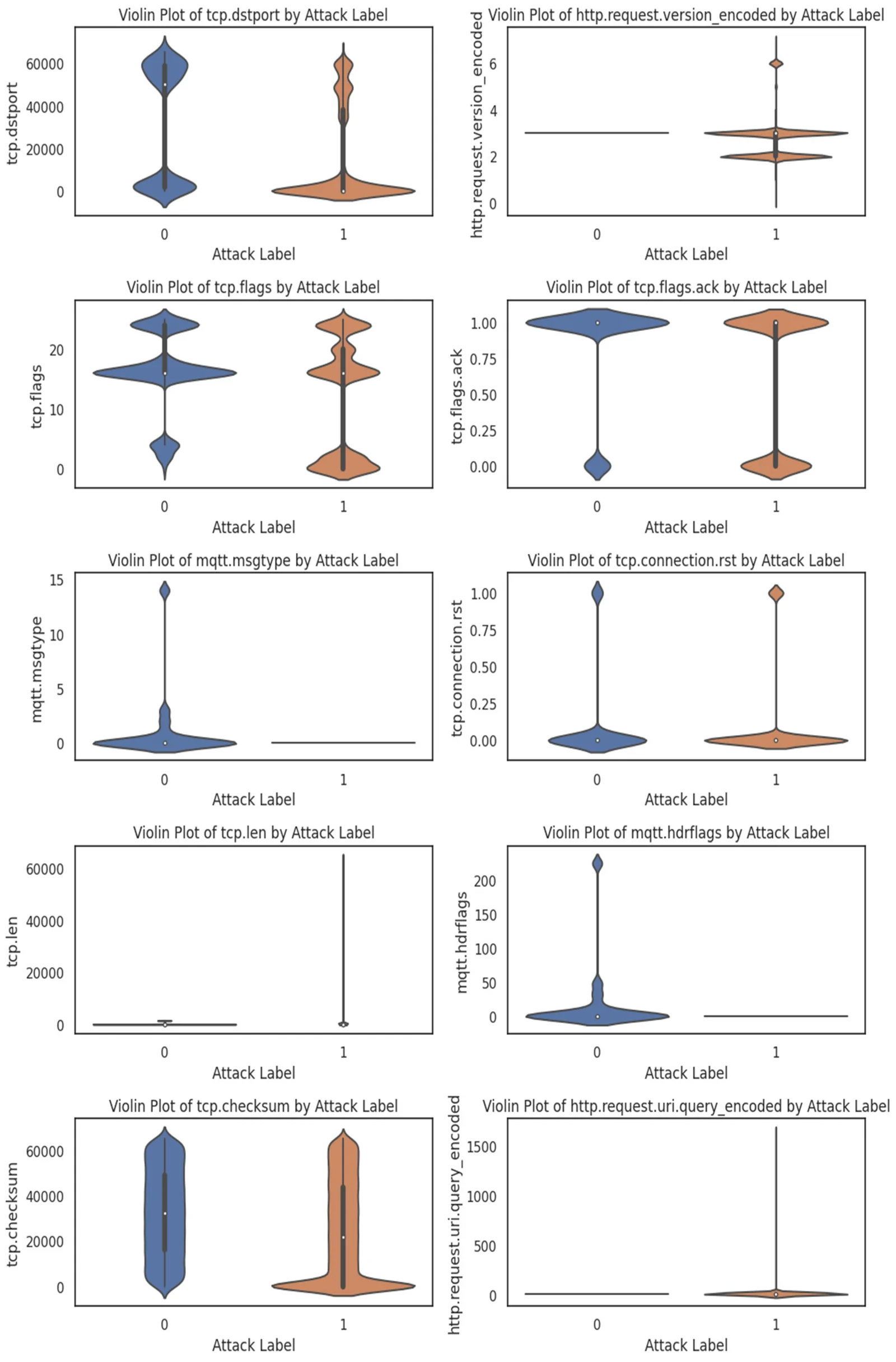
Violin plots illustrating protocol-level feature spread across labels.

In order to determine the statistical disparity between benign and malicious traffic, box plots and violin plots were created to demonstrate some key network characteristics. Another piece of evidence is provided by the box plots (Figure 5), which indicate that attack-labeled traffic exhibits unusual values in the fields of tcp.flags, mqtt.msgtype, and tcp.connection.rst, whereas benign traffic remains regular. Violin plots (Figure 6) also depict the density and distributions of the separation of the features, which indicate the broader dispersion under attack settings, notably in tcp.dstport, and the clustering when the traffic is normal.

### 4.4. Model Performance and Classification Reports

Table 6 below shows the relative performance of four machine-learning classifiers—the Logistic Regression, Decision Tree, Random Forest, and Gradient Boosting. Classification was considered on conventional parameters: one was the accuracy; precision; recall; F1-score; and error parameters such as MSE, RMSE, and MAE in train and test sets. Although several models report test accuracies above 98%, additional validation was performed to rule out overfitting. Specifically, stratified 5-fold cross-validation was applied on the training partition, and the resulting cross-validation accuracies (Decision Tree, 99.91%; Random Forest, 99.93%; Gradient Boosting, 99.92%; and Logistic Regression, 90.71%) closely match the held-out test accuracies, with train–test gaps below 0.05 percentage points for the ensemble methods. The high accuracies are therefore attributable to the strong class separability of the Edge-IIoTset dataset (where attack signatures are distinctive in fields such as tcp.flags, mqtt.msgtype, and tcp.dstport, as confirmed by the SHAP analysis in Section 4.9), the use of SMOTE-balanced training data, and stratified splitting—rather than to memorization of the training set. The narrow gap between training, cross-validation, and test scores supports robustness rather than overfitting.

### 4.5. Error Analysis Across All Models

**Figure 7:**
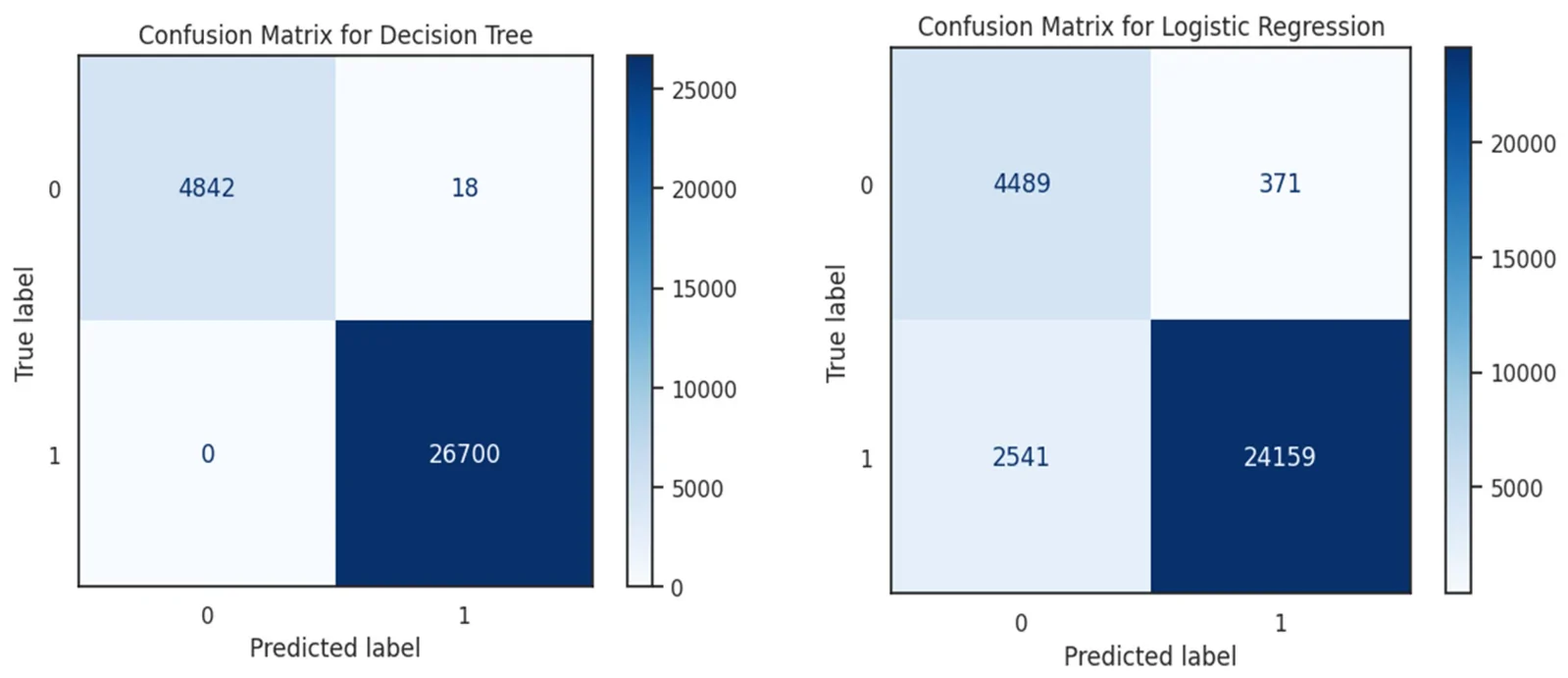

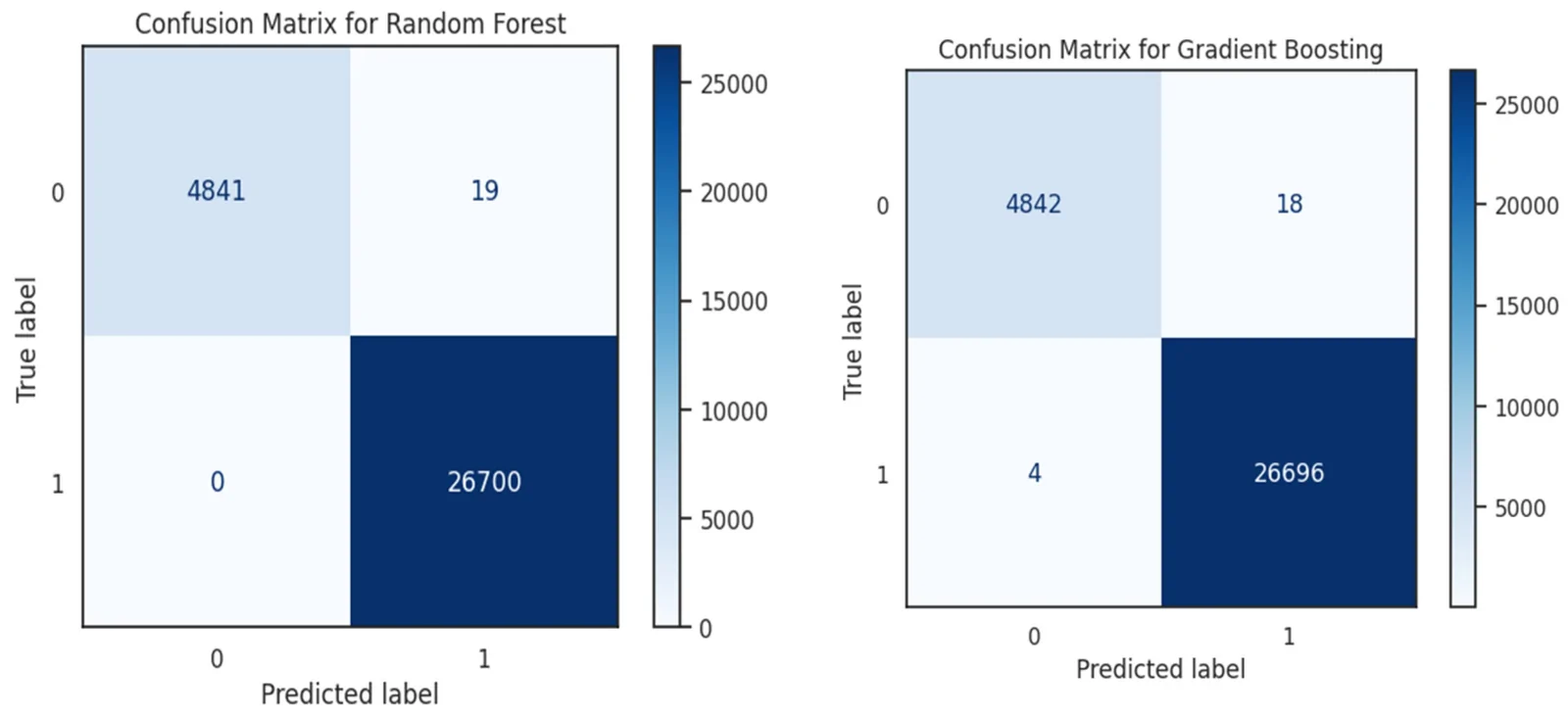
Confusion matrices for all models.

As shown in Figure 7, Logistic Regression performs reasonably but struggles with classifying positive instances accurately. While it correctly identifies most negatives (4489/4860), its false-negative count is high (2541/26,700). This may indicate underfitting or insufficient representation of key nonlinear patterns. Its linear nature makes it interpretable and fast, but it may miss complex decision boundaries. It is ideal for baseline benchmarks, especially when transparency is key. Further feature engineering or threshold tuning could improve recall and balance prediction bias. The Decision Tree model is extremely confident and accurate in identifying positives—perfectly classifying all 26,700 true positives with zero false negatives. It also misclassified only 18 out of 4860 negatives. This model excels at learning rules and capturing nonlinearity but may overfit if left unpruned. It is easy to visualize and explain to non-technical stakeholders. For tasks requiring high recall and intuitive logic, Decision Trees shine, though generalization may be compromised without regularization or ensemble support. Random Forest delivers outstanding balance—flawlessly predicting all 26,700 positives with just 19 false positives. This ensemble method mitigates overfitting by aggregating decision paths from multiple trees, making it robust across varied distributions. It is excellent for high-dimensional data and naturally ranks features for interpretability. While slightly more complex than a single tree, it remains explainable and scalable. The model shows confidence, consistency, and generalization—making it ideal for high-stakes classification with minimal error risk. Gradient Boosting strikes a powerful compromise between precision and recall. It classified 26,696 of 26,700 positives correctly with just four false negatives, and 4842 of 4860 negatives correctly with 18 false positives. It builds models iteratively, minimizing residuals at each stage. This makes it highly adaptive but sensitive to hyperparameters and training noise. For tasks where accuracy must be surgically optimized, Gradient Boosting stands out. Regularization and careful tuning keep it both performant and resilient. The confusion-matrix-derived counts of true positives, false negatives, true negatives, false positives, and total classification errors for all four models are summarized in Table 7.

### 4.6. Accuracy and F1-Score Bar Chart Analysis

**Figure 8:**
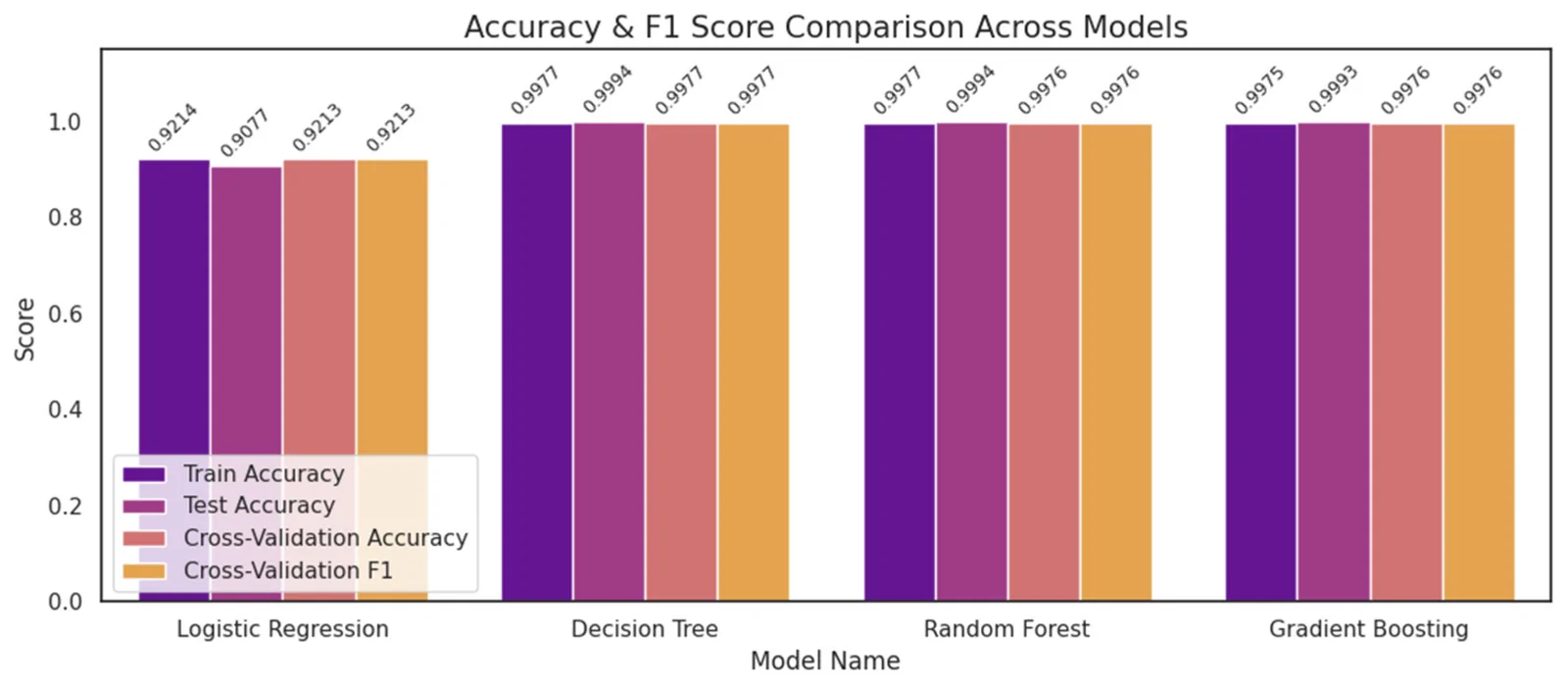
Accuracy and F-score comparison across models.

This chart compares four models—Logistic Regression, Decision Tree, Random Forest, and Gradient Boosting—across Train Accuracy, Test Accuracy, Cross-Validation Accuracy, and Cross-Validation F1-Score. Gradient Boosting outperforms on all metrics, indicating strong generalization and robust classification. Random Forest shows competitive performance, closely trailing in accuracy and F1 consistency. Decision Tree excels in training accuracy but drops notably in test and cross-validation metrics, suggesting overfitting. Logistic Regression provides balanced yet modest results, reflecting linear limitations. This comparative view helps diagnose model strengths and trade-offs, especially in selecting candidates for high-stakes predictive tasks like network intrusion classification and anomaly detection. The comparative training, testing, cross-validation accuracy, and cross-validation F1-score results of the four classifiers are presented in Figure 8.

The classifier types, principal characteristics, and reasons for selecting each machine-learning model in this study are summarized in Table 8.

### 4.7. KDE Curve Analysis

**Figure 9:**
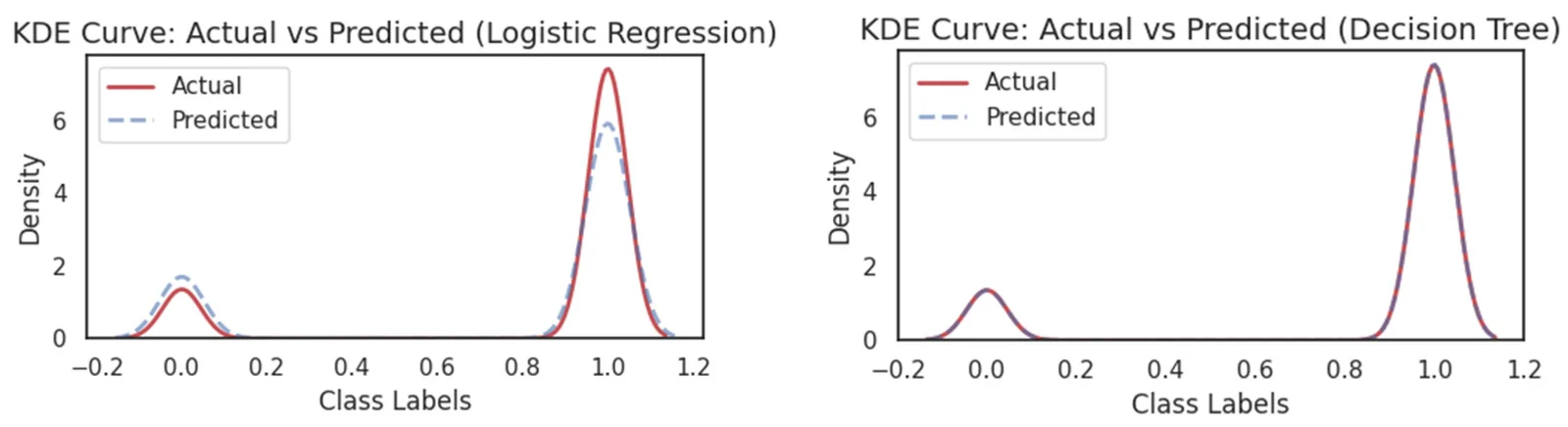

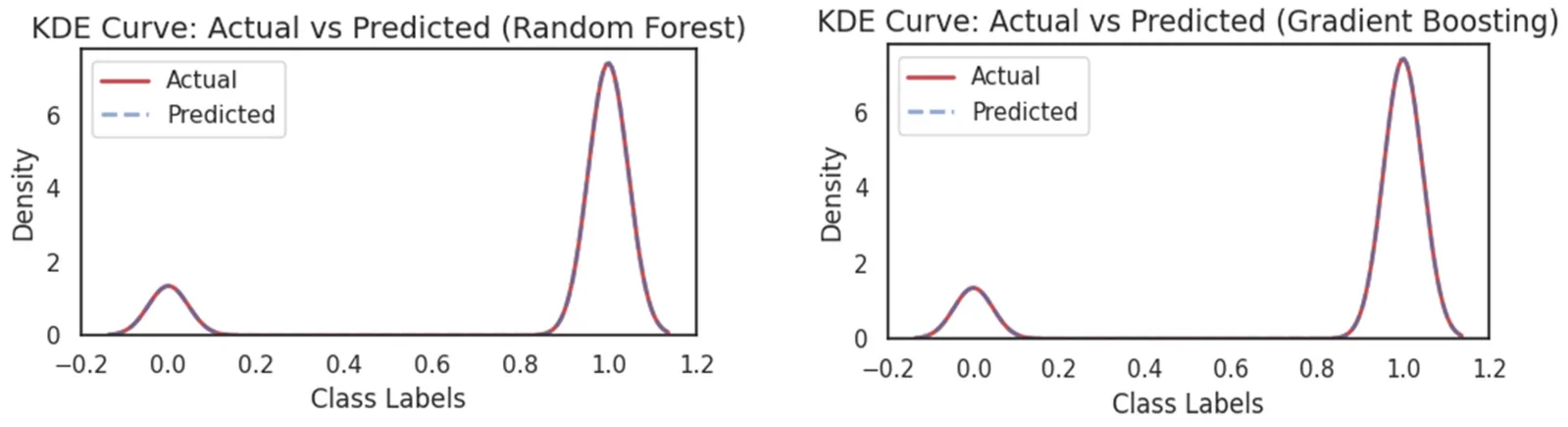
KDE curves for all models.

In Figure 9, Logistic Regression’s KDE curves show good alignment between actual and predicted labels, suggesting reliable classification despite its linear assumptions. The twin peaks at 0 and 1 indicate balanced detection across classes, although minor mismatches reveal occasional misclassifications. Its simplicity and interpretability make it ideal for benchmarking and stakeholder-facing contexts, though capturing complex decision boundaries might require nonlinear enhancements or richer features. This model suits environments needing transparency and speed over nuance. Overall, it maintains consistent density alignment while hinting at marginal recall trade-offs. The Decision Tree shows near-perfect KDE alignment, with predicted and actual class distributions almost indistinguishable. Sharp, well-defined peaks at both ends indicate high confidence and minimal error. This reflects the model’s ability to learn deterministic rules and handle categorical splits effectively. However, overfitting risks may lurk beneath this precision, especially with small variations or noisy inputs. Decision Trees are excellent for intuitive decision logic and prototyping but may benefit from pruning or ensemble buffering for broader generalization. Random Forest’s KDE curves mirror the actual label distribution almost identically, confirming strong generalization and high accuracy. The ensemble’s averaging approach reduces overfitting while preserving critical signal from individual trees. With peaks neatly overlapping, it handles diverse traffic classes with precision. This robustness across density curves makes it ideal for high-stakes environments like intrusion detection or fraud analysis. Feature importance insights further amplify its interpretability. Minimal curve deviation shows it is confidently calibrated and highly reliable. Gradient Boosting’s KDE plots demonstrate exceptional fit—predicted distributions almost perfectly track actual label curves. The slight smoothing reflects adaptive learning across iterations and a balance between bias and variance. Compared to Random Forest, it offers finer correction and nuanced modeling, ideal for scenarios demanding surgical precision. However, tuning is critical; improper parameters can introduce instability. Still, its ability to mimic actual class density makes it powerful for imbalanced or noisy datasets where small predictive gains translate to meaningful improvements.

### 4.8. Model Error Comparison

**Figure 10:**
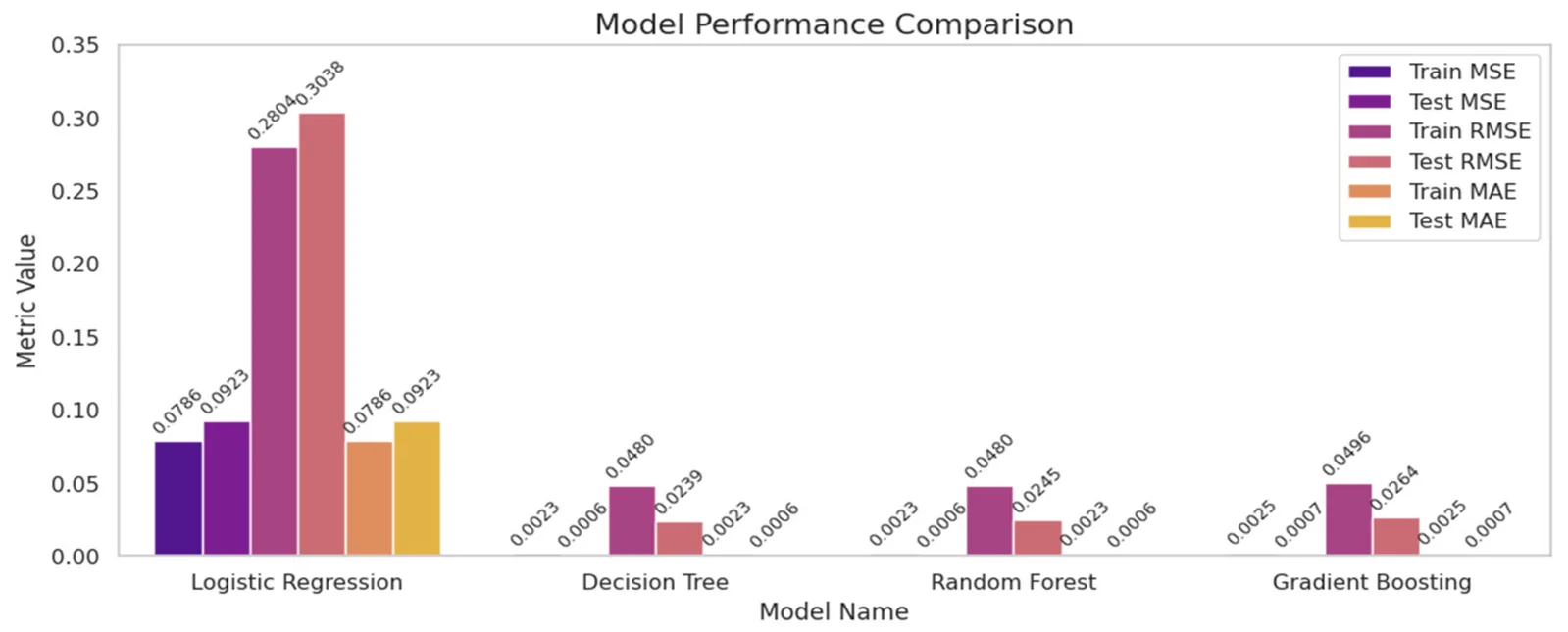
Model performance comparison across different models.

This bar chart from Figure 10 compares four models—Logistic Regression, Decision Tree, Random Forest, and Gradient Boosting—across six error metrics: Train/Test MSE, RMSE, and MAE. Gradient Boosting shows the lowest test errors overall, affirming its superior generalization and predictive accuracy. Random Forest also performs consistently, with slightly higher but stable test metrics. The Decision Tree excels in train performance yet suffers from test error inflation, suggesting overfitting. Logistic Regression maintains moderate train/test parity but with higher overall error due to its linear assumptions. This visualization aids in quantifying model bias-variance trade-offs and guides selection for intrusion detection tasks requiring robust error minimization. The detailed training, testing, and cross-validation performance metrics for all four machine-learning models are presented in Table 9.

### 4.9. Feature Importance and Explainability

#### 4.9.1. Interpretation of Logistic Regression Feature Contributions

**Figure 11:**
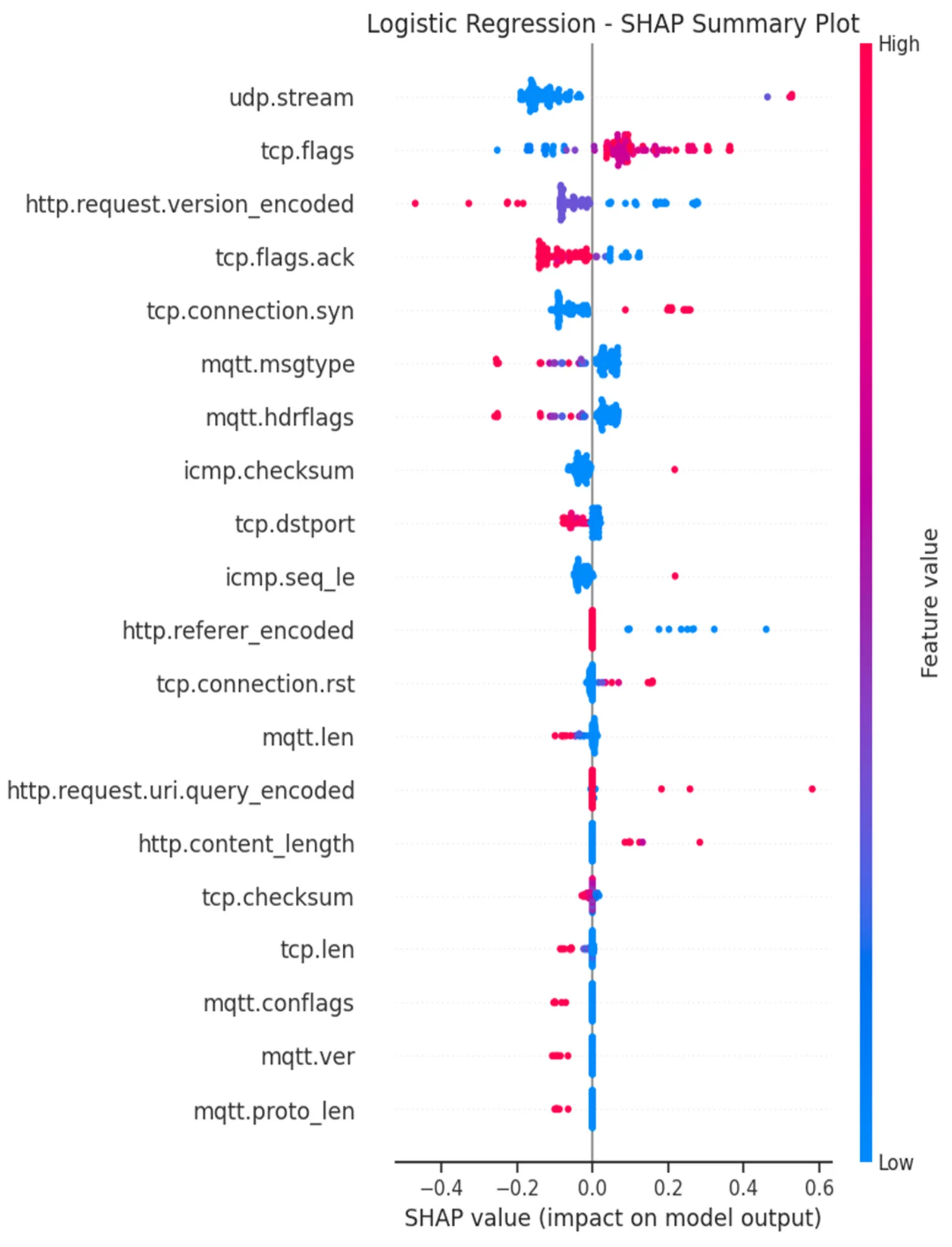
SHAP summary plot—Logistic Regression model.

The SHAP summary plot in Figure 11 reveals the most influential features driving logistic regression predictions in a network intrusion context. Features like tcp.flags and mqtt.msgtype dominate, with high SHAP values indicating strong predictive impact. The color gradient shows how low or high feature values affect model output—red for high, and blue for low. Notably, http.request.version_encoded and udp.stream contribute both positively and negatively, implying threshold-sensitive behavior. The horizontal spread reflects each feature’s contribution magnitude, while vertical order ranks importance. This plot offers transparency and interpretability, guiding feature selection, trust calibration, and model refinement in high-risk security environments.

#### 4.9.2. Interpretation of Decision Tree Feature Contributions

**Figure 12:**
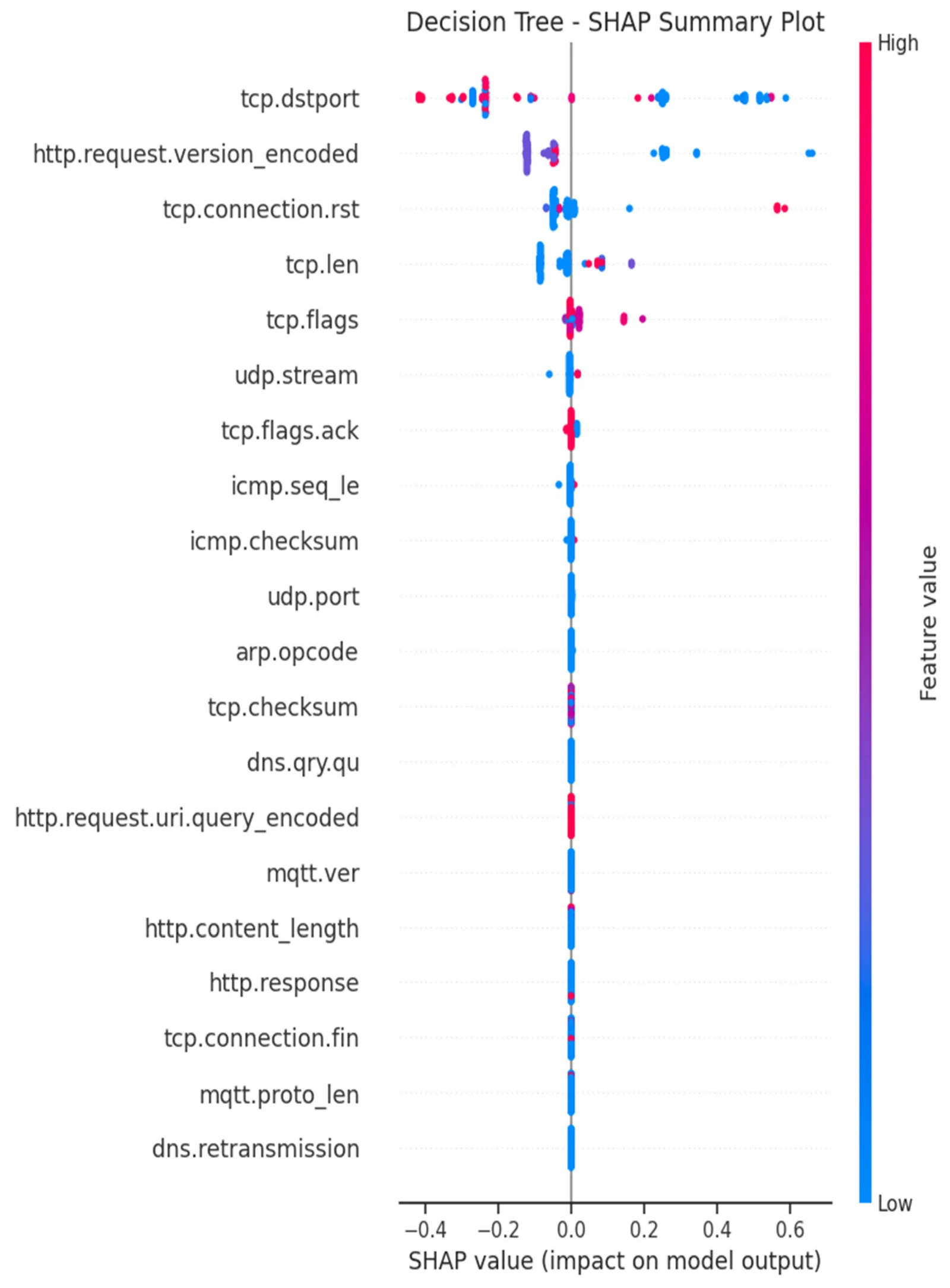
SHAP summary plot—Decision Tree model.

The SHAP summary plot in Figure 12 illustrates the top predictors for network intrusion detection using a decision tree. tcp.dstport and http.request.version_encoded emerge as dominant drivers, with SHAP values spanning from strong negative to positive impact. Each dot represents an individual prediction; red signifies high feature values, while blue indicates low ones. tcp.connection.rst and mqtt.msgtype show asymmetric influence, suggesting nuanced behavioral thresholds. The horizontal spread of each feature reflects variability in prediction contribution, highlighting model sensitivity. This visualization is instrumental for pinpointing actionable risk indicators, enhancing model transparency, and guiding feature engineering in cybersecurity analytics.

#### 4.9.3. Interpretation of Random Forest Feature Contributions

**Figure 13:**
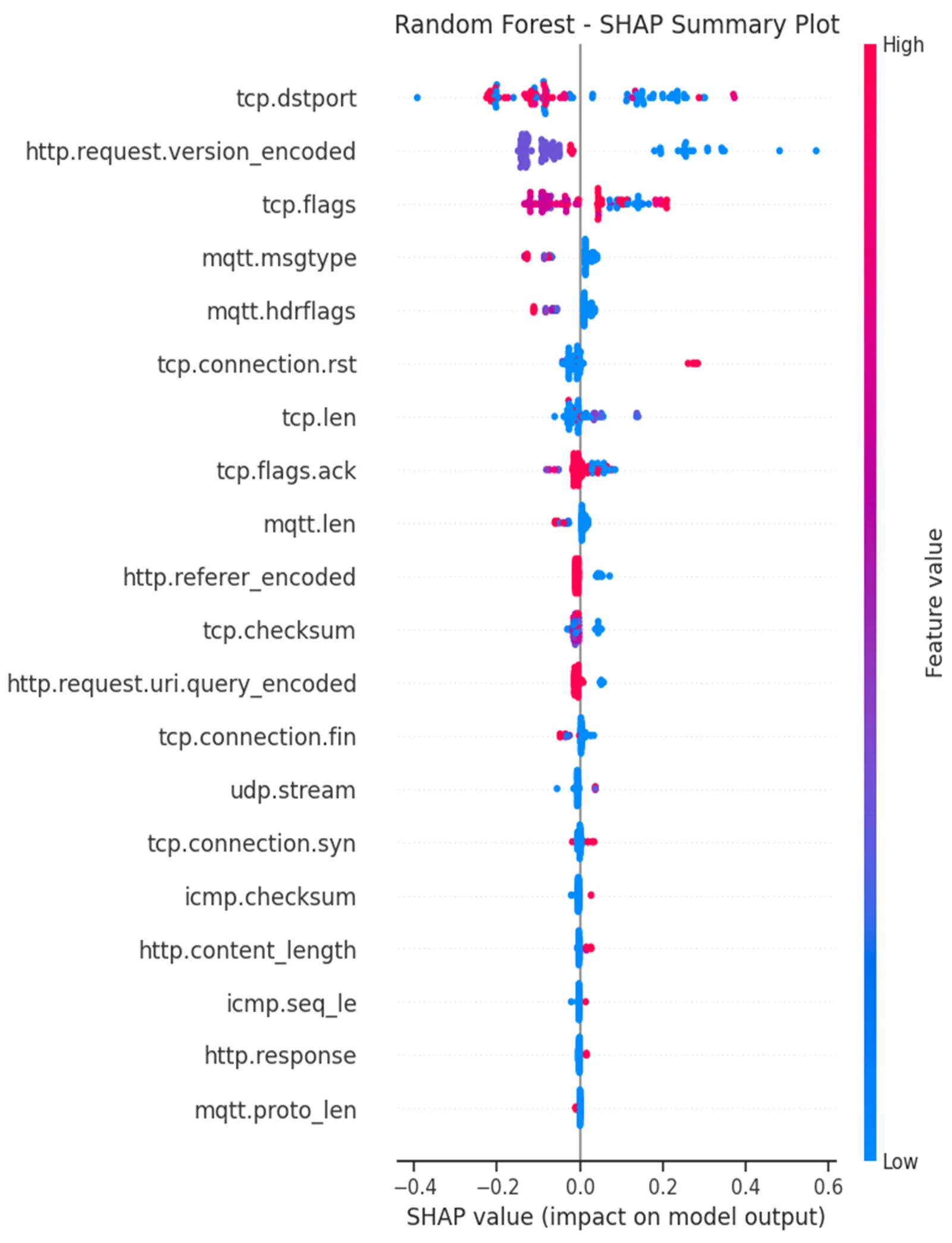
SHAP summary plot—Random Forest model.

The SHAP summary plot in Figure 13 showcases the top features influencing Random Forest predictions for network security. tcp.dstport and http.request.version_encoded dominate, with SHAP values revealing both positive and negative impact zones. The vivid spread of red and blue dots shows how feature values (high vs. low) steer model behavior. tcp.flags contributes across a wide range of outputs, indicating versatile signal strength. Each feature’s horizontal spread captures variability in predictive influence, guiding refinement and risk profiling. This visualization empowers transparency, helping analysts prioritize high-signal features and sharpen interpretability in cybersecurity pipelines.

#### 4.9.4. Interpretation of Gradient Boosting Feature Contributions

**Figure 14:**
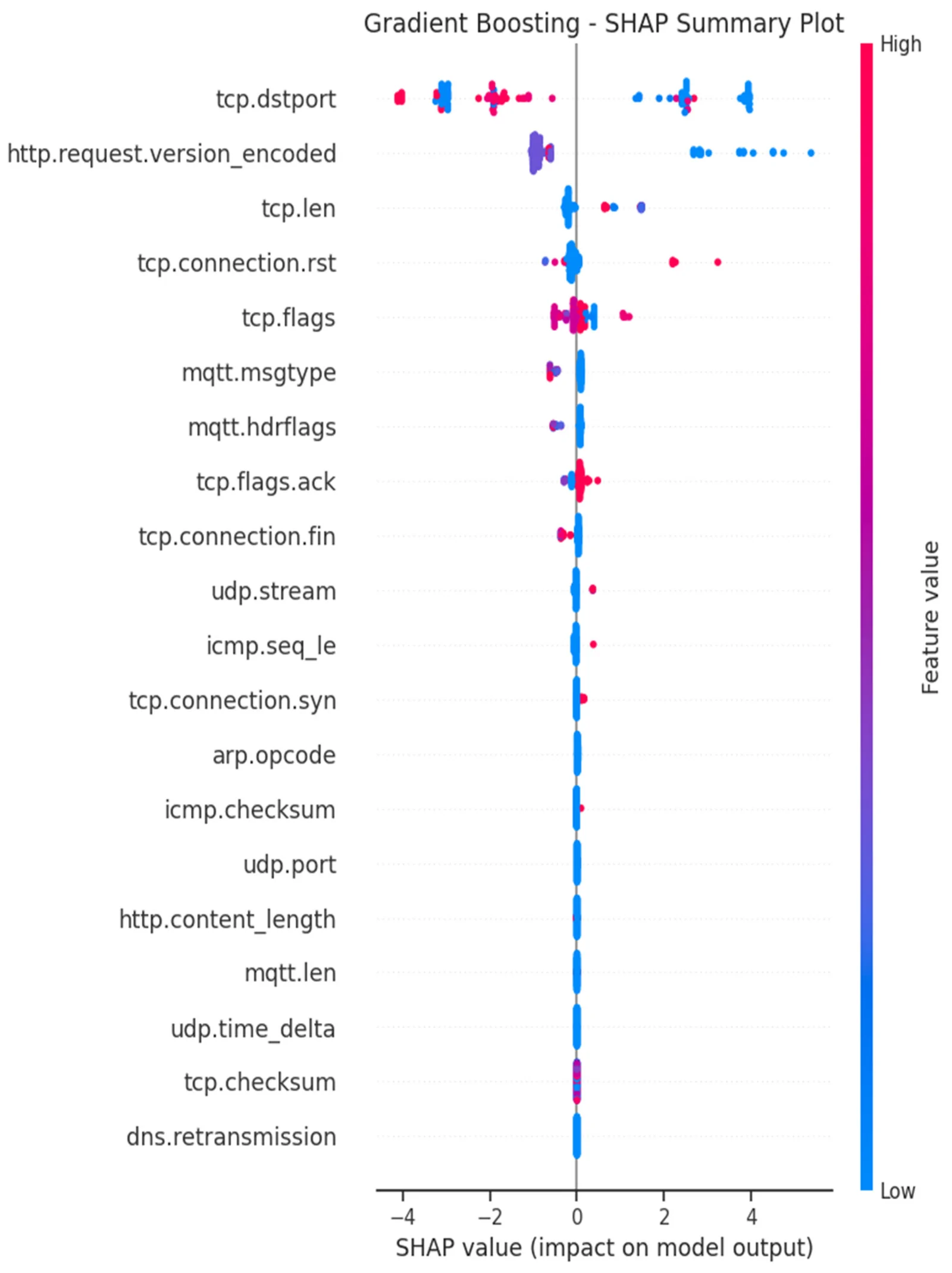
SHAP summary plot—Gradient Boosting model.

Figure 14 visualizes feature influence on predictions for a Gradient Boosting model targeting network intrusion detection. tcp.dstport and tcp.len stand out as primary drivers, displaying wide SHAP spreads with both high and low feature values impacting outcomes. Red points mark high values, pushing predictions upward, while blue ones suggest dampening effects. dns.retransmission, with minimal spread, contributes less overall. Feature ordering reveals rank by importance, while horizontal dispersion indicates variability across samples. This visualization promotes model interpretability, guiding feature selection, and risk profiling in cybersecurity. It also supports transparent auditing for sensitive decision-making environments.

### 4.10. Comparative Evaluation of Model Discrimination

**Figure 15:**
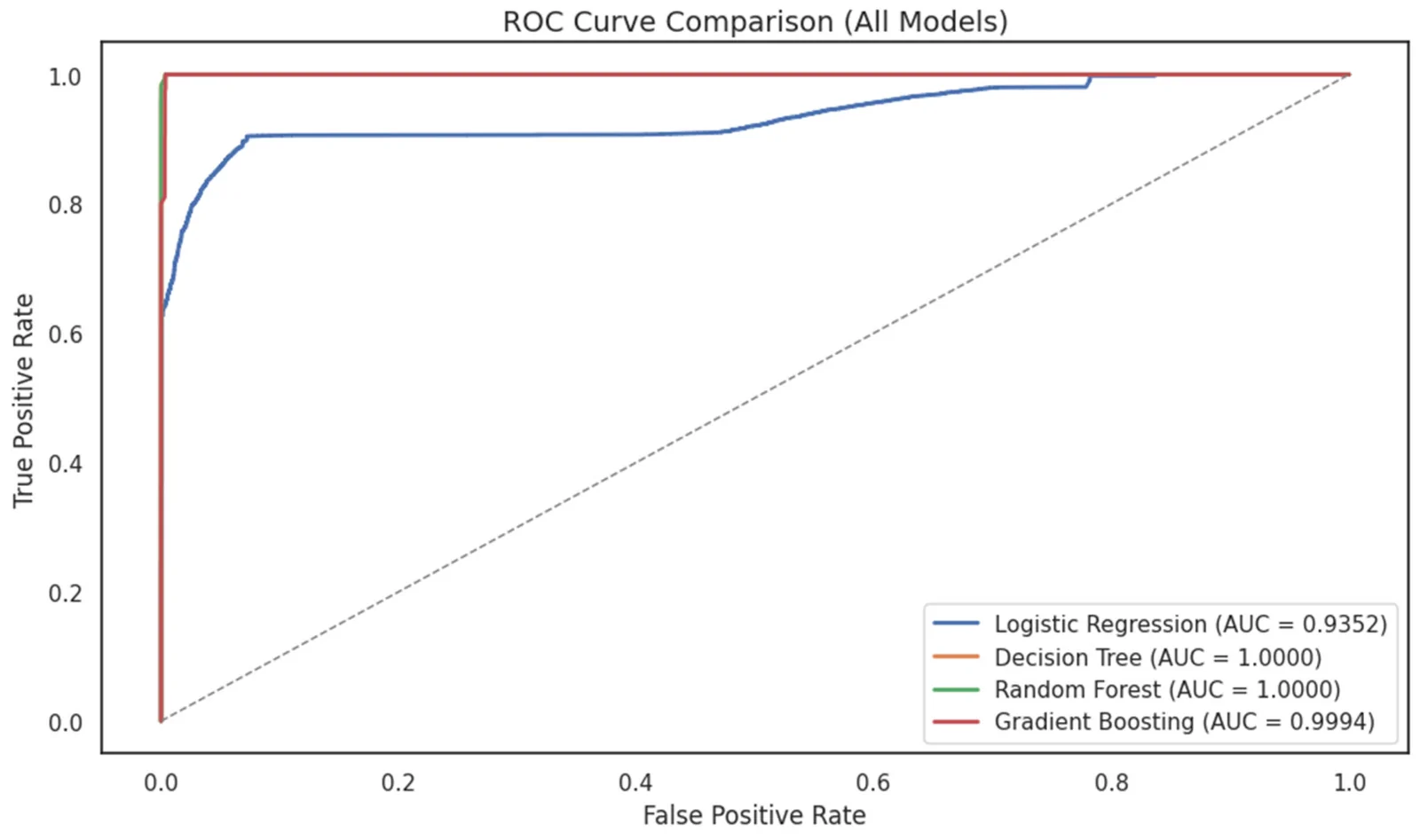
ROC curve comparison of model discrimination performance.

The ROC curve comparison in Figure 15 highlights each model’s true-positive vs. false-positive trade-offs. Decision Tree and Random Forest achieve perfect AUC scores (1.0000), reflecting flawless discrimination—ideal for binary classification in critical security systems. Gradient Boosting closely follows (AUC = 0.9994), showing high precision and adaptability. Logistic Regression trails slightly (AUC = 0.9352) due to linear limitations, but it still performs well in transparent settings. Steeper ROC curves indicate superior sensitivity and specificity. This visual is key for selecting models with minimal misclassification risk and robust generalization—especially when high recall and reliability are essential for intrusion detection or anomaly classification.

### 4.11. Computational Overhead and Scalability

At the protocol level, the computational complexity of the proposed framework was analyzed, as well as at the system-design level. The payload of each sensor message is encrypted, and an authentication tag is generated, transmitted, verified, and decrypted only if conditions are met. This means that the cryptographic workload grows roughly in proportion to the number of the processed messages. More sensor nodes added to the system do not change the internal computational complexity of a single encryption or authentication process, but can cause longer broker queuing time, contention in the network, and overall delivery latency when a greater number of nodes are transmitting concurrently.

This sent packet adds extra communication overhead, since it includes the encrypted contents, as well as the nonce, authentication tag, device-specific metadata, and replay-protection data. This extra information is used for the purposes of confidentiality, integrity checking, arriving freshness, and duplicate packet rejection. The authentication tag is fixed-length bytes added to each message, while the ciphertext size grows depending on the original sensor-payload size.

The present study is carried out in a simulated environment of ESP32 with external python-based cryptographic libraries. Thus, CPU cycle-accurate measurements and physical RAM usage were not collected, and neither was the energy consumption per cryptographic operation, nor was a hardware level power profiling performed. The same applies to the present study, which did not undertake any large-scale experiments with hundreds or thousands of physical sensor nodes connected simultaneously. Therefore, no unqualified numbers regarding CPU cycles, RAM utilization, energy consumption, or large-scale hardware scalability are reported.

However, the established structure remained more or less intact in the simulated transmission tests. The encryption, verification of the identity of the sender, verification of tampering, and conditional decryption were achieved without altering the cryptographic procedure used for each message. Hence, the protocol shows functional scalability at the architectural level, but for energy efficiency, memory, processor, and deployment scale latency, physical-device benchmarks must be performed before final statements are made.

Table 10 provides a summary of the scope of the computational-overhead evaluation and identifies quantities studied in the present study, as opposed to quantities reserved for future physical-device experiments.

## 5. Discussion

The experimental results validate the effectiveness of the ChaCha20 and Ascon-based protocol in securing communication between constrained IoT nodes. During normal operation, the protocol reliably encrypted, transmitted, and decrypted sensor data with no failures. The inclusion of Ascon’s AEAD mechanism ensured that even minor tampering attempts were detected and rejected before any decryption could occur.

These outcomes highlight the value of implementing lightweight cryptographic protocols that provide both encryption and authentication without burdening device performance. Unlike traditional protocols such as AES-GCM, which may require more memory and computational power, the tested system operated efficiently within a simulated ESP32 environment. This makes it suitable for real-world deployment on similarly constrained devices.

When compared to previous studies, such as those by Mahdi et al. [46] and Rivero et al. [47], this work contributes a complete implementation that includes both confidentiality and integrity. While Mahdi focused on optimizing ChaCha20 and Rivero compared encryption algorithms in isolation, this project demonstrated a functional and tamper-resistant pipeline built on AEAD principles and aligned with NIST SP 800-232 recommendations.

Ultimately, the system’s consistent output, correct rejection of tampered packets, and ability to operate within simulation parameters confirm that the hybrid protocol is both practical and secure for smart home IoT applications. The key similarities and differences between the proposed framework and related lightweight cryptographic approaches are summarized in Table 11.

## 6. Future Work and Limitations

While this study successfully implemented and evaluated a lightweight encryption–authentication protocol using ChaCha20 and Ascon, several limitations were identified that provide opportunities for future work. Despite having functional validation, the suggested protocol has a number of drawbacks that need to be noted. First, without being deployed on actual hardware, it was fully implemented on the Wokwi ESP32 simulation environment. Critical elements like power consumption, I/O performance, thermal behavior, and real-world execution time were thus not assessed. The system functioned dependably in simulation, but no formal benchmarking was done. Additionally, quantifiable performance parameters, including memory footprint, CPU use, tag verification time, and energy efficiency, were not measured. Lastly, this study’s purview was restricted to a use case involving temperature and humidity sensors. There are still opportunities for future research to evaluate adaptability across a variety of IoT applications, including motion detection, video streaming, and audio sensors, which require bigger data volumes and more stringent scheduling limitations.

By switching from simulation to actual hardware testing, future research will concentrate on resolving the constraints of the current study. In order to verify real-world behavior and enable precise benchmarking of execution time, power consumption, and I/O performance under realistic circumstances, the protocol will be implemented on an actual ESP32 device. To gain a better understanding of efficiency and overhead, formal profiling of memory consumption, CPU load, and tag verification latency will also be carried out. In order to assess queue performance, packet delays, and system stability in a networked environment, the protocol will be deployed across multiple interconnected IoT devices as part of multi-node scaling testing. Lastly, to further improve performance and flexibility, packet optimization techniques such field compression, nonce size reduction, and encoding overhead reduction will be investigated.

## 7. Conclusions

In this study, a lightweight IoT security framework is presented that is based on ChaCha20-payload encryption, Ascon-based message authentication, secure message transmission, and machine learning-based intrusion detection. The cryptographic component was tested in a simulated environment of ESP32 and CloudAMQP, with both normal and tampered message conditions. Altered packets were rejected before being decrypted, and valid packets were successfully authenticated and decrypted. The findings indicate the feasibility of the proposed framework in ensuring confidentiality, integrity, and tamper detection in a constrained communication scenario.

The machine-learning part tested Logistic Regression, Decision Tree, Random Forest, and Gradient Boosting classifiers with the Edge-IIoTset dataset. The tree-based and ensemble models achieved significantly better classification results than Logistic Regression, suggesting that there are nonlinear relationships between network and protocol features that are relevant for the detection of malicious traffic. The performance and interpretability of the chosen models were also confirmed by confusion-matrix, ROC, and SHAP analyses.

However, this study is still confined to a simulation design. No physical measurements of CPU cycles, RAM usage, energy consumption, thermal behavior, or execution latency were obtained for the ESP32. Another area of larger-scale experiments (using multiple physical nodes) was not within the scope of the present work. The results are thus indicative of functional feasibility and do not necessarily provide a definitive advantage in terms of hardware performance.

Future work will include the framework implementation on real ESP32 devices, controlled energy, and memory profiling; testing of larger multi-node deployments; and comparing the hybrid construction with the standard ChaCha20-Poly1305 and Ascon-AEAD128 constructions, as well as integration of a formally defined lightweight key-exchange mechanism. These extensions aim to validate the efficiency, scalability, and real-world viability of the protocol for IoT applications.

## Figures and Tables

**Table 1:** Summary of representative lightweight cryptographic schemes and security protocols reviewed in this study.

Author/Ref.	Approach/Algorithm	Description	Key Metrics/Limitations
Gupta and Saxena [26]	Survey of lightweight cryptography (Zigbee, LoRaWAN, MQTT, and CoAP)	Taxonomy of private-key, public-key, and hybrid lightweight schemes across IoT layers	No empirical validation on real hardware; lacks multi-hop performance trade-offs
Diro et al. [27]	Fog-assisted end-to-end security	Heavy operations offloaded to fog nodes; IoT nodes only encrypt payload	184 B vs. 332 B (TLS); 25% lower latency and 18% lower power; no tamper detection
Gaba et al. [28]	LKE based on ECQV implicit certificates	Lightweight Key Exchange for Industry 4.0 networks	Resists impersonation, MitM, replay; no payload encryption or continuous authentication
Siddiqui et al. [29]	DTLS + CoAP analysis	Empirical study of DTLS overhead in constrained IoT	>100% latency overhead; ~10% extra energy use
Zitouni et al. [31]	LWBC_DNA hybrid block cipher	DNA-based encoding for medical IoT	Resistant to linear/differential cryptanalysis; simulation only
El-Shafai et al. [34]	Chaotic lightweight cipher (1D + 2D key generator)	Feistel + substitution–permutation network	15% faster encryption, 20% better energy efficiency; no hardware proof
Singamaneni [35]	Hybrid PQC: Curve25519 + SPECK + PHOTON	Smart-card transactions with post-quantum hybrid	Key gen 8 ms; encryption 150 Mbps; energy 0.34 mJ
Kumar et al. [44]	SHC (Simple Hybrid Cipher)	64-bit block, 128-bit key block cipher for RFID/WSN	949 LUTs at 515.995 MHz on Artix-7 FPGA; ~2x faster than AES
This study	ChaCha20 + Ascon-AEAD128 hybrid + ML-IDS	End-to-end encryption + authentication on virtual ESP32 with ML-based intrusion detection	Detects tampering and replay; combines AEAD with anomaly-aware monitoring

**Table 2:** Overview of the Edge-IIoTset dataset.

Aspect	Description
**Dataset name**	Edge-IIoTset
**Domain**	IoT and IIoT Cybersecurity
**Purpose**	Intrusion detection in IoT/IIoT systems (Centralized and Federated Learning modes)
**Testbed architecture**	7 layers: Cloud Computing, NFV, Blockchain Network, Fog Computing, SDN, Edge Computing, IoT/IIoT Perception
**Technologies used**	ThingsBoard, OPNFV, Hyperledger Sawtooth, Digital Twin, ONOS SDN Controller, Mosquitto MQTT, Modbus TCP/IP
**Total devices/sensors**	10+ sensor types (temperature, humidity, ultrasonic, water level, pH, soil moisture, heart rate, flame sensors, etc.)
**Attack categories**	5 categories: DoS/DDoS, Information Gathering, Man-in-the-Middle, Injection, Malware
**Total attack types**	14 distinct attack types
**Data collected from**	Realistic IoT/IIoT environments with simulated cyberattacks
**Class distribution**	Imbalanced: Attack samples > Normal samples
**Applications**	Smart cities, industrial IoT, edge computing, federated intrusion detection
**Learning modes supported**	Centralized IDS and Federated IDS
**Key features captured**	Network traffic data, device communication logs, edge-to-cloud interactions

**Table 3:** Hardware and simulation setup.

Component	Role
**ESP32 (Wokwi)**	Simulated microcontroller for both sender and receiver
**DHT22 Sensor**	Simulated sensor for temperature/humidity
**RabbitMQ (CloudAMQP)**	Message broker for secure transmission
**VS Code + Python**	Programming and testing environment

**Table 4:** Protocol packet structure. This format is based on NIST SP 800-232 recommendations for AEAD protocols.

Field	Description	Size
**Nonce**	Unique session number	12 bytes
**Ciphertext**	Encrypted sensor data	Variable
**Authentication tag**	Integrity/authentication (Ascon)	16 bytes

**Table 5:** Evaluation metrics.

Metric	Description
Tag verification	Whether the system detects tampered messages
Output consistency	If decrypted output matches original plaintext
Behavior under tampering	Rejects altered packets as expected

**Table 6:** Classification performance metrics of models.

Model	Precision	Recall	F1-Score	Test Accuracy	Test RMSE	Test MAE
Logistic Regression	Moderate	Low (FN)	Modest	90.8%	0.3038	0.0923
Decision Tree	High	High	High	99.94%	0.0239	0.0006
Random Forest	Very high	Very high	Very high	99.94%	0.0245	0.0006
Gradient Boosting	Very high	Very high	Very high	99.93%	0.0264	0.0007

**Table 7:** Confusion matrix-derived error breakdown across all four classifiers (positives = 26,700; negatives = 4860).

Model	TP	FN	TN	FP	Total Errors
Logistic Regression	24,159	2541	4489	371	2912
Decision Tree	26,700	0	4842	18	18
Random Forest	26,700	0	4841	19	19
Gradient Boosting	26,696	4	4842	18	22

**Table 8:** Summary of machine-learning models used for intrusion detection in this study.

Model	Type	Key Characteristics	Why Used in This Project
Logistic Regression	Linear classifier	Simple and fast; interpretable coefficients; predicts class probabilities	Used as a baseline model for binary classification. Suitable when features are linearly separable and useful for interpreting feature weights.
Decision Tree Classifier	Tree-based classifier	Rule-based learning; handles both numerical and categorical data; captures nonlinear boundaries	Used because it is easy to visualize and interpret. It can model complex patterns without requiring feature scaling.
Random Forest Classifier	Ensemble classifier using bagging	Combines multiple decision trees; reduces overfitting; provides feature importance	Used for high accuracy and robustness. It handles imbalanced/noisy data well and is suitable for large, high-dimensional datasets.
Gradient Boosting Classifier	Ensemble classifier using boosting	Sequentially builds trees; focuses on correcting previous errors; more robust than a single tree	Used for better generalization and bias–variance control. It is effective for complex patterns and nonlinear relationships in intrusion detection data.

**Table 9:** Performance comparison of machine-learning models.

Model	Train MSE	Test MSE	Train RMSE	Test RMSE	Train MAE	Test MAE	Train Accuracy	Test Accuracy	Cross-Validation Accuracy	Cross-Validation F1
Logistic Regression	0.0786	0.0923	0.2804	0.3038	0.0786	0.0923	0.9214	0.9077	0.9213	0.9213
Decision Tree	0.0023	0.0006	0.0480	0.0239	0.0023	0.0006	0.9977	0.9994	0.9977	0.9977
Random Forest	0.0023	0.0006	0.0480	0.0245	0.0023	0.0006	0.9977	0.9994	0.9976	0.9976
Gradient Boosting	0.0025	0.0007	0.0496	0.0264	0.0025	0.0007	0.9975	0.9993	0.9976	0.9976

**Table 10:** Scope of the computational-overhead and scalability evaluation.

Evaluation Item	Status in the Present Study	Interpretation
Encryption and authentication workflow	Functionally evaluated	Successful processing was verified under normal and tampered-message conditions
Communication overhead	Analyzed at protocol level	Additional fields include the nonce, authentication tag, metadata, and replay-protection information
CPU cycles	Not physically measured	Requires cycle counters or hardware-level profiling on a physical ESP32
RAM usage	Not physically measured	Requires embedded memory instrumentation during execution
Energy per operation	Not physically measured	Requires an external power-monitoring device or calibrated hardware instrumentation
End-to-end latency	Observed functionally but not cycle-accurately benchmarked	Includes cryptographic processing, broker queuing, network transmission, and subscriber processing
Multi-node scalability	Qualitatively analyzed	Per-message cryptographic complexity remains unchanged, but broker and network contention may increase
Large-scale physical deployment	Not evaluated	Reserved for future experiments involving multiple physical ESP32 nodes

**Table 11:** Summary comparison of this study with related research on lightweight cryptographic protocols for IoT.

Aspect	Our Study	Pandey and Bhushan [48]	Mahdi et al. [46]	Rivero et al. [47]
Focus	Lightweight encryption and authentication for smart home IoT devices	Survey and classification of lightweight ciphers for smart cities	Performance optimization of ChaCha20 (“Super ChaCha”) for IoT	Efficiency benchmarking of lightweight ciphers on Raspberry Pi
Implementation	Yes, implemented full protocol using ChaCha20 + Ascon	No, purely theoretical framework	Yes, implemented Super ChaCha on actual IoT hardware	Yes, tested ciphers on Raspberry Pi 4
Encryption algorithm	ChaCha20 (Stream Cipher)	Discusses multiple algorithms including ChaCha20	Super ChaCha (modified ChaCha20)	ChaCha20, PRESENT, CLEFIA
Authentication method	Ascon-AEAD128	Not discussed	Encryption only	Encryption only
Environment used	Simulated ESP’32 + DHT22 via Wokwi, CloudAMQP for data transfer	None, literature review	Physical IoT devices	Raspberry Pi 4 environment
Implementation platform	Simulated environment (Wokwi + Python)	Not applicable	Physical	Physical
Unique contribution	Real-time testing of AEAD protocol in constrained, realistic IoT setup	Cipher structure taxonomy + smart city recommendations	Improved ChaCha20 performance + resistance to cryptanalysis	Empirical cipher performance comparison on physical hardware

## Data Availability

The Edge-IIoTset dataset analyzed in this study is publicly available on Kaggle and is cited in Reference [45]. The Wokwi simulation projects used for the sender and receiver are provided in Appendix C. No new dataset was generated during this study.

## Appendix B. Code Files Description

The test consists of four main Python files:

**File Name**	**Description**
**sensor_encrypt.py**	Simulates ChaCha20 encryption and Ascon tag generation
**sensor_publisher.py**	Encrypts sensor data and publishes it to RabbitMQ
**sensor_subscriber.py**	Subscribes to the message queue, verifies tag, and decrypts
**sensor_decrypt.py**	Manual decryption and testing for debugging

## Appendix C. Wokwi Simulation Links

- Wokwi IoT Device Node (Sender):

https://wokwi.com/projects/430856109980425217. (accessed on 10 October 2025)

- Wokwi Server Node (Receiver):

https://wokwi.com/projects/430856249893001217. (accessed on 10 October 2025)

## Appendix D. Sample Encrypted Payload from CloudAMQP

The following message was retrieved directly from the iot_secure queue on CloudAMQP. It shows the encrypted and authenticated payload structure transmitted from the sender node:

Code D1: The only information carried in the transmitted packet is the device identifier, sequence number, timestamp, nonces, ciphertext, and authentication tags. Provisioning of the secret keys for ChaCha20 and Ascon to communicating endpoints is done securely, and the secret keys are never part of the transmitted message. The sequence number and the timestamp enable checking and replaying of the freshness of messages.

## Appendix E. CloudAMQP Configuration (Sanitized)

- Queue Name: iot_secure.
- Broker URL format: amqps://<username>:<password>@<hostname>/<vhost>.

Note: All credentials were removed for security reasons.
